# Essential Role of Latrophilin-1 Adhesion GPCR Nanoclusters in Inhibitory Synapses

**DOI:** 10.1523/JNEUROSCI.1978-23.2024

**Published:** 2024-04-29

**Authors:** Daniel Matúš, Jaybree M. Lopez, Richard C. Sando, Thomas C. Südhof

**Affiliations:** ^1^Department of Molecular and Cellular Physiology, Stanford University School of Medicine, Stanford, California 94305; ^2^Department of Pharmacology, Vanderbilt Brain Institute, Vanderbilt University, Nashville, Tennessee 37240; ^3^Hughes Medical Institute, Stanford University School of Medicine, Stanford, California 94305

**Keywords:** adhesion GPCRs, inhibitory synapses, latrophilin, synapse formation, synapses, synaptic adhesion

## Abstract

Latrophilin-1 (Lphn1, aka CIRL1 and CL1; gene symbol *Adgrl1*) is an adhesion GPCR that has been implicated in excitatory synaptic transmission as a candidate receptor for α-latrotoxin. Here we analyzed conditional knock-in/knock-out mice for Lphn1 that contain an extracellular myc epitope tag. Mice of both sexes were used in all experiments. Surprisingly, we found that Lphn1 is localized in cultured neurons to synaptic nanoclusters that are present in both excitatory and inhibitory synapses. Conditional deletion of Lphn1 in cultured neurons failed to elicit a detectable impairment in excitatory synapses but produced a decrease in inhibitory synapse numbers and synaptic transmission that was most pronounced for synapses close to the neuronal soma. No changes in axonal or dendritic outgrowth or branching were observed. Our data indicate that Lphn1 is among the few postsynaptic adhesion molecules that are present in both excitatory and inhibitory synapses and that Lphn1 by itself is not essential for excitatory synaptic transmission but is required for some inhibitory synaptic connections.

## Significance Statement

Previously, the adhesion GPCRs Latrophilin-2/Latrophilin-3 have been shown to mediate excitatory, but not inhibitory, synapse assembly onto discreet dendritic compartments of hippocampal pyramidal neurons. Here we now show that Latrophilin-1 is targeted to both excitatory and inhibitory hippocampal synapses. Unexpectedly, we find that Latrophilin-1 is selectively essential for directing inhibitory synaptic connections to the neuronal soma. Our work supports a model by which latrophilins are widely used as organizers of synaptic connectivity that act on a subcellular level. In the light of recent findings connecting haploinsufficiency of Latrophilin-1 to a plethora of neurodevelopmental and neuropsychiatric disorders, our study contributes to our understanding of the molecular mechanisms of latrophilins that need to be targeted in order to address various pathologies.

## Introduction

Synapses are fundamental units of neuronal networks. While postsynaptic neurotransmitter reception, presynaptic vesicle release, and synaptic plasticity have been studied intensively (Kandel, 2001; Südhof, 2013; Bailey et al., 2015; Basu and Siegelbaum, 2015; Chen and Gouaux, 2019; Scott and Aricescu, 2019), mechanisms of synapse formation are still poorly understood. Recent insights into this area came from the study of synaptic adhesion molecules, such as neurexins, neuroligins, or adhesion GPCRs (aGPCRs; Cao and Tabuchi, 2017; Südhof, 2017, 2018; Sanes and Zipursky, 2020; Fuccillo and Pak, 2021; Gomez et al., 2021; Boxer and Aoto, 2022; Cortés et al., 2023). aGPCRs are characterized by a variety of N-terminal adhesion domains, a GPCR autoproteolysis-inducing (GAIN) domain that mediates autoproteolysis, a canonical 7 transmembrane receptor domain typical for all GPCRs, and a rather long cytoplasmic region (reviewed in Hamann et al., 2015; Vizurraga et al., 2020; Rosa et al., 2021; Einspahr and Tilley, 2022; Liebscher et al., 2022; Seufert et al., 2023). This domain combination places aGPCRs at the intersection of adhesion and signal transduction, although the precise functions of most aGPCRs are unclear. Multiple aGPCRs, including brain angiogenesis inhibitors (BAIs), latrophilins, and cadherin EGF LAG-repeat seven-transmembrane receptors (CELSRs), are thought to contribute to synapse formation (Bolliger et al., 2011; Najarro et al., 2012; Stephenson et al., 2013; Kakegawa et al., 2015; Sigoillot et al., 2015; Zhu et al., 2015; Martinelli et al., 2016; Anderson et al., 2017; Thakar et al., 2017; Tu et al., 2018; Sando et al., 2019; Wang et al., 2020, 2021; Zhou et al., 2021; Li et al., 2022; Shiu et al., 2022; Aimi et al., 2023; Freitas et al., 2023).

Three latrophilins are expressed in mice (protein names Lphn1–3; gene symbols *Adgrl1–3*; Sugita et al., 1998; Ichtchenko et al., 1999), with primary transcripts that are subject to extensive alternative splicing (Sugita et al., 1998; Knierim et al., 2019; Ovando-Zambrano et al., 2019). Latrophilins are postsynaptic receptors (Anderson et al., 2017; Sando et al., 2019) that contain N-terminal lectin- and olfactomedin-like domains that bind to presynaptic teneurins (Silva et al., 2011; Boucard et al., 2014) and Flrts (O’Sullivan et al., 2012), respectively. Intracellularly, latrophilins interact with SHANK proteins (Kreienkamp et al., 2000; Tobaben et al., 2000; Wang et al., 2024), which are components of postsynaptic density–protein scaffolds (Naisbitt et al., 1999). Interestingly, the latrophilin-mediated synapse formation requires G-protein coupling (Sando and Südhof, 2021), suggesting that synaptic adhesion complexes constitute sites of active signaling. In the hippocampus, Lphn2 and Lphn3 mediate excitatory synapse formation onto different subcellular regions of CA1 pyramidal neurons (Sando et al., 2019), whereas in the cerebellum Lphn2/3 act redundantly in parallel-fiber synapse formation (Zhang et al., 2020). Such “molecular codes” are also employed by other synaptic adhesion molecules, such as neurexins or neuroligins (Cao and Tabuchi, 2017; Südhof, 2017, 2018; Sanes and Zipursky, 2020; Fuccillo and Pak, 2021; Gomez et al., 2021; Boxer and Aoto, 2022; Cortés et al., 2023).

The progress in studying Lphn1 was hindered by the lack of specific antibodies and by the behavioral abnormalities of Lphn1 constitutive knock-out (KO) mice (Tobaben et al., 2002; Vitobello et al., 2022). A recent study revealed that loss-of-function mutations of Lphn1 (*ADGRL1*) cause major neurodevelopmental disorders in humans and synaptic impairments in mice (Vitobello et al., 2022). Here, we generated conditional KO (cKO) mice that allow the localization of the endogenous protein by virtue of a knocked-in myc epitope tag. We show using stimulated emission depletion (STED) microscopy that Lphn1 forms nanoclusters in both excitatory and inhibitory synapses of hippocampal neurons. However, in contrast to Lphn2/3, Lphn1 specifically mediates inhibitory synapse formation onto the cell soma. Neuronal morphology was unchanged after Lphn1 deletion. These findings expand on how latrophilins define synaptic specificity, be it excitatory versus inhibitory or the spatial restriction of synapses to subcellular regions.

## Materials and Methods

### Animal procedures

All mice housed at Stanford University were weaned at 20–21 d of age and housed in groups of 2–5 on a 12 h light/dark cycle with access to food and water *ad libitum*. All procedures conformed to National Institutes of Health Guidelines for the Care and Use of Laboratory Mice and were approved by the Stanford Animal Use Committees (Administrative Panel for Laboratory Animal Care/Institutional Animal Care and Use Committee). Mice housed at Vanderbilt University were weaned at 18–21 d of age and housed in groups of 2–5 on a 12 h light/dark cycle with food and water *ad libitum*. Vanderbilt Animal Housing Facility: All procedures conformed to National Institutes of Health Guidelines for the Care and Use of Laboratory Mice and were approved by the Vanderbilt University Administrative Panel on Laboratory Animal Care. Ai14 mice were obtained from Jackson Laboratory (7914) and were crossed to L1f cKO mice to obtain homozygous L1f cKO/homozygous Ai14 reporter. Baf53b-Cre mice (Actl6b-Cre) were obtained from Jackson Laboratory (27826). Mice were bred on a hybrid background to avoid penetrance of background mutations in inbred mouse strains. Mice of either sex were used in all experiments.

### Generation of Lphn1 cKO mice

Lphn1 cKO mice were generated from European Conditional Mouse Mutagenesis (EUCOMM) ES cells [Adgrl1^tm2a(EUCOMM)Hmgu^, parental cell line JM8A3.N1 (B6N)] at the Janelia Research Campus. Founder mice were crossed to FLP mice [B6N.Cg-Tg(ACTFLPe) 9205Dym/CjDswJ #19100] to remove the selection cassette and LacZ reporter, generating a conditional floxed exon 4 [start, 84,649,628; end, 84,649,737 (GRCm39)] of Lphn1 (Adgrl1, ENSMUSG00000013033). Lphn1 cKO/FLP mice were crossed to C57/Bl6J (Jackson Laboratory, 664) to remove the FLP allele, and heterozygous Lphn1 cKO nonlittermates subsequently crossed to homozygosity. Lphn1 cKO mice are available via Jackson Laboratory (035185). The following primers were used to genotype Lphn1 cKO alleles:

Reaction 1: 5′-AGGCATCCTTATCCATGGAG-3′, 5′-TGCTATGGAGTGCAGAGACT-3′, 5′-CTCCTACATAGTTGGCAGTG-3′ [WT 374 bps, cKO 259 bps (before FLP recombination)/531 bps (after FLP recombination)]
Reaction 2: 5′- CTTGATCCAGTACACCTGTG-3′, 5′- GGCTCTGAAGGACTTTAGCA-3′ (WT 237 bps, cKO 317 bps)

Myc-tagged Lphn1 cKO mice were custom generated at Janelia Research Campus. In brief, exon 3 [start, 84,645,449; end, 84,645,662 (GRCm39)] of the Lphn1 gene (Adgrl1, ENSMUSG00000013033) was duplicated, with one duplicated exon containing a 2xmyc tag inserted after the signal peptide, and the second exon containing a myc-SNAP tag following the signal peptide. Preferred frt sites flank the neomycin selection cassette and the exon containing myc-SNAP tag. Nonpreferred frt3 sites flank the exon containing a 2xmyc tag and neomycin cassette. Both duplicated exons 3 are flanked by loxP1 sites to allow conditional deletion. Founder mice were crossed to B6N.Cg-Tg(ACTFLPe)9205Dym/CjDswJ #19100, and a PCR-based strategy was used to discriminate between FLP-mediated recombination events to select for 2xmyc-exon 3 containing Lphn1 mice. myc-Lphn1 mice identified as positive via PCR were subsequently crossed to C57/Bl6J to remove the FLP allele. Myc-Lphn1 cKO mice are available via Jackson (035181). The following primers were used for genotyping the myc-Lphn1 allele:

Reaction 1: 5′- CATAGATgttaacGGATCCACC-3′, 5′- CCGGTACTGTAAGCTTTGTAG-3′ (mutant 222 bps)
Reaction 2: 5′- GACACACAGTTGTGACTGAC-3′, 5′- GCATCGCAGATCTTGTCATC-3′ (WT 359 bp, mutant 527 bp)
Reaction 3: 5′- GCACGTCACACTGACATTGT-3′, 5′- TCTGTGAGCTCCTACCTGAA-3′ (WT 271 bp, mutant 367 bp)

### Antibodies

Concentrations of antibodies that have been used for different experimental approaches in this study (immunohistochemistry, IHC; immunocytochemistry, ICC; immunoblot, IB) were as follows: Avidin-Alexa Fluor 488 (Invitrogen, catalog #S32354) 1:1,000 (IHC); rabbit anti-c-myc (Sigma-Aldrich, catalog #C3956) 1:500 (ICC, IHC), 1:1,000 (IB); mouse anti-β-actin (Sigma-Aldrich, catalog #A1978) 1:5,000 (IB); chicken anti-MAP2 (EnCor Biotechnology, catalog #CPCA-MAP2) 1:2,000 (ICC); guinea pig anti-vGLUT1 (Synaptic Systems, catalog #135304) 1:1,000 (ICC); mouse anti-pan-SHANK (NeuroMab, catalog #75-089) 1:1,000 (ICC); guinea pig anti-vGAT (Synaptic Systems, catalog #131005) 1:1,000 (ICC); mouse anti-Gephyrin (NeuroMab, catalog #75-444) 1:1,000 (ICC); mouse anti-Homer1 (Synaptic Systems, catalog #160011) 1:1,000 (ICC); rabbit anti-NeuN (Merck Millipore, catalog #ABN78) 1:1,000 (ICC); mouse anti-GAD67 (Merck Millipore, catalog #MAB5406) 1:1,000 (ICC); donkey anti-rabbit IRDye800CW (LI-COR Biosciences, catalog #926-32213) 1:10,000 (IB); donkey anti-mouse IRDye680LT (LI-COR Biosciences, catalog #926-68022) 1:10,000 (IB); goat anti-chicken Alexa Fluor 405 (Thermo Fisher Scientific, catalog #A48260) 1:1,000 (ICC); goat anti-chicken Alexa Fluor 546 (Thermo Fisher Scientific, catalog #A11040) 1:1,000 (ICC); goat anti-rabbit Alexa Fluor 405 (Thermo Fisher Scientific, catalog #A31556) 1:1,000 (ICC); goat anti-rabbit Alexa Fluor 546 (Thermo Fisher Scientific, catalog #A11035) 1:1,000 (ICC); goat anti-mouse Alexa Fluor 488 (Thermo Fisher Scientific, catalog #A11029) 1:1,000 (ICC); goat anti-mouse Alexa Fluor 647 (Thermo Fisher Scientific, catalog #A21236) 1:1,000 (ICC); goat anti-guinea pig Alexa Fluor 647 (Thermo Fisher Scientific, catalog #A21450) 1:1,000 (ICC); goat anti-rabbit STAR RED (Abberior, catalog #STRED-1002) 1:250 (ICC); goat anti-guinea pig STAR ORANGE (Abberior, catalog #STORANGE-1006) 1:500 (ICC); goat anti-mouse STAR 460L (Abberior, catalog #ST460L-1001) 1:500 (ICC).

### Lentiviral production

HEK293T cells (ATCC) were grown in a 37°C and 5% CO_2_ atmosphere in DMEM (Invitrogen, catalog #11995065) + 10% fetal bovine serum (FBS (Sigma-Aldrich, catalog #F0926)). Lentiviral constructs expressing NLS-GFP-ΔCre or NLS-GFP-Cre driven by the human synapsin promoter were described previously (Kaeser et al., 2011). In this study, similar constructs from the Südhof Laboratory have been used (NLS-tdTomato-ΔCre or NLS-tdTomato-Cre under the influence of the human synapsin promoter or NLS-GFP-ΔCre or NLS-GFP-Cre under the influence of the ubiquitin promoter). Lentiviral packaging was achieved by transfecting 10.8 µg of the respective shuttle vectors in addition to helper plasmids (3 µg of pRSV-REV, 7.35 µg of pMDLg/pRRE, and 3.84 µg of vesicular stomatitis virus G-protein) into 75 cm^2^ of 40–50% confluent HEK293T culture using a calcium phosphate approach. DNA was diluted in 450 µl of ddH_2_O and 50 µl of 2.5 M CaCl_2_ solution was added. Then the DNA-CaCl2 mix was added dropwise into an equal volume of 2× HEPES-buffered saline (274 mM NaCl, 10 mM KCl, 1.5 mM Na2HPO4, 12 mM glucose, 42 mM HEPES, adjust pH to 7.05 using NaOH) while gently vortexing the tube. After 20 min of incubation at room temperature, the transfection mix was briefly flicked and added dropwise to the cells. After 24 h, the medium was exchanged to neuron growth medium (see below, Primary hippocampal cultures, for the components), and after 48 h, the virus-containing medium was filtered through 0.22 µm polyethersulfone membranes (GenClone, catalog #25-243), aliquoted and stored at −80°C for application in culture experiments. For each virus batch, the lowest necessary infectious titer was determined to infect >95% of cultured neurons and corresponding quantities were added in all culture experiments. For lentiviral concentration, viral conditioned media were centrifuged at 5,000 × *g* for 5 min to pellet cellular debris, filtered and ultracentrifuged at 55,000 × *g* for 1.5 h through a sucrose cushion. Pellets were resuspended in MEM (Invitrogen catalog #51200038) at 1/500 of the initial volume, aliquoted and stored at −80°C.

### Primary hippocampal cultures

Hippocampus dissection from postnatal day 0 (P0) pups (gender unknown) was performed in ice-cold HBSS [Hanks balanced salt solution (Sigma-Aldrich, catalog #H2387-10X1L), 1 mM HEPES (pH 7.3), 4 mM NaHCO3], and the tissue was digested in papain solution [100 µl papain suspension (Worthington Biochemical, catalog #LS003127) in 5 ml HBSS] for 30 min at 37°C. The tissue was then washed 2× in HBSS and 1× in plating medium [5% FBS (Sigma-Aldrich, catalog #F0926), B27 supplement (Invitrogen, catalog #17504044), 0.4% glucose, and 2 mM glutamine diluted in 1× MEM (Invitrogen, catalog #51200038)] and thoroughly triturated. After filtration through a 70 µm cell strainer (Falcon, catalog #352350), cells were suspended in plating medium either in 6-well plates coated with 0.05 M poly-D-lysine (Sigma-Aldrich, catalog #P7280) diluted in 0.05 M borate buffer (for protein extraction) or in 24-well plates containing zero thickness glass coverslips [Assistant, catalog #01105209, coated with Matrigel (Corning, catalog #356235)]. On DIV1 80% of the medium was exchanged to the neuron growth medium [5% FBS (Sigma-Aldrich, catalog #F0926), B27 supplement (Invitrogen, catalog #17504044), and GlutaMAX (Invitrogen, catalog #35050061) in Neurobasal A (Invitrogen, catalog #10888022)]. On DIV3 and DIV8, 50% of the medium was exchanged for a neuron growth medium containing 4 µM cytosine arabinofuranoside (Santa Cruz Biotechnology, catalog #221454A). Lentivirus infections were performed during the media change on DIV3 (if not stated otherwise) and sparse calcium phosphate transfections on DIV8. Neuronal cultures were kept in a 37°C and 5% CO_2_ atmosphere and analyzed on DIV14–16.

### Protein collection, SDS-PAGE, and IB

To extract total protein, we washed neuronal cultures twice with ice-cold PBS and lysed for 20 min with a freshly prepared RIPA buffer [50 mM Tris–HCl (pH 8), 1 mM EDTA, 150 mM NaCl, 0.5% sodium deoxycholate, 0.1% SDS, 1% Triton X-100, 1 mM PMSF, and cOmplete Protease Inhibitor Cocktail (Sigma-Aldrich, catalog #11873580001)] on ice, under light agitation. The samples were then spun down in a tabletop centrifuge (precooled to 4°C) at 15,000 g for 20 min, and pellets were discarded. Concentrations of protein samples were measured with the Micro BCA Protein Assay Kit (Life Technologies, catalog #23235), using a BSA standard curve. Equal amounts of protein were diluted in a 5× sample buffer (50 mM Tris–HCl, pH 6.8, 10% SDS, 40% glycerol, 100 mM DTT, and bromophenol blue), loaded on 4–20% Mini-PROTEAN TGX Precast Gels (Bio-Rad Laboratories) together with Xpert 2 Prestained Protein Marker (GenDEPOT, catalog #P8503), and run at a 25 mA/gel constant current. The Trans-Blot Turbo Transfer System and Buffer (Bio-Rad Laboratories, catalog #1704150) was used following manufacturer's instructions to perform a semi-wet transfer onto a 0.45 µm Amersham Protran nitrocellulose membrane (Sigma-Aldrich, catalog #GE10600002). For blocking, membranes were incubated for 1 h at 22°C in 5% nonfat dry milk/TBS (20 mM Tris–HCl, pH 7.4, 150 mM NaCl). Membranes were then incubated overnight at 4°C in a primary antibody diluted in 5% milk/TBST (TBS + 0.05% Tween), washed 3 × 5 min with TBST, incubated for 45 min at 22°C with corresponding secondary antibodies diluted in TBST containing 0.01% SDS, washed for 5 × 5 min with TBST, and imaged using a LI-COR Odyssey system.

### Quantitative RT-PCR

Primary hippocampal cultures were generated from P0 Lphn1 cKO (EUCOMM) mice as described above and infected at DIV1 with lentiviral ubiquitin-driven GFP-CRE or GFP-ΔCRE. At DIV14, total mRNA was isolated using the Qiagen RNeasy Mini Kit (catalog #74104) and mRNA concentration and purity via A260/280 ratio measured using a NanoDrop (Thermo Fisher Scientific). qPCR measurements were performed with VeriQuest Probe One-Step qRT-PCR Master Mix (Affymetrix, catalog #75700) and a 10 ng mRNA template using an Applied Biosystems 7900HT apparatus using predesigned PrimeTime qPCR probe assays (Integrated DNA Technologies). The following probes were used: *Mm* Lphn1, Mm.PT.58.42048881 (exon 2–3); Mm.PT.58.6572453 (exon 4–5); Mm.PT.58.30545738 (exon 8–10); and Mm.PT.58.10233084 (exon 21–23), and *Mm* Gapdh, Mm.PT.39a.1. Respiratory quotient (RQ) was calculated via RQ = 2^−^^[^*^Ct^*^(^^target, CRE^^)^^−^*^Ct^*^(^^target, ΔCRE^^)]^^−^^[^*^Ct^*^(^^Gapdh, CRE^^)^^−^*^Ct^*^(^^Gapdh, ΔCRE^^)]^.

### Sparse transfections of neuron cultures

In order to analyze neuronal morphology, neurons need to be visualized in their entirety without overlapping dendrites or axons. Therefore, sparse transfection using a calcium phosphate-based approach was performed to deliver a CMV promoter-driven EGFP plasmid into individual neurons. Per well in a 24-well plate, the following mixture was prepared: 0.5 µg of DNA, 1.5 µl of 2.5 M CaCl_2_, and ddH_2_O to a total volume of 15 µl. While gently vortexing a tube containing an equivalent volume of 2× BBS, pH 7 (50 mM BES, 280 mM NaCl, 1.5 mM Na_2_HPO_4_), the DNA/calcium phosphate mixture was added dropwise and left to precipitate at room temperature for 15 min. Seventy-five percent (750 µl) of neuron growth medium was taken off the cultured neurons and kept at 37°C. Neurons were then washed 3× with prewarmed MEM (Invitrogen, catalog #51200038) in the form of 75% media exchanges. After the final wash, 500 µl of MEM was kept in each well. The transfection mix was briefly vortexed before 30 µl were added dropwise per well, followed by 10 min of incubation at 37°C and 5% CO_2_. Thereafter, three 75% media exchanges for fresh prewarmed MEM were performed, leaving 100 µl per well after the last wash. 500 µl of saved preconditioned medium and 400 µl of fresh neuron growth medium were added to each well, and the culture was put back into a 37°C and 5% CO_2_ atmosphere.

### ICC of cultured cells

For surface labeling, cells were washed 1× in bath solution (see below, Culture electrophysiology, for the composition) and incubated for 20 min at room temperature in primary antibody. After being washed 3× with a bath solution, neurons were then fixed in 4% PFA (Electron Microscopy Sciences, catalog #15714) diluted in bath solution for 20 min at room temperature, washed 3× in PBS, blocked for 60 min in 5% goat serum (Jackson ImmunoResearch Laboratories, 005000121) in PBS, and stained with appropriate secondary antibody, diluted in 5% goat serum in PBS. Cells were then washed 4× with PBS, fixed a second time in 4% PFA diluted in PBS for 5 min at room temperature, and washed 3× in PBS before permeabilization and subsequent staining. If no surface labeling was performed, cells were washed 1× in a bath solution, fixed for 20 min at room temperature in 4% PFA diluted in a bath solution, and washed 3× in PBS. Cells were permeabilized/blocked for 60 min at room temperature in 0.2% Triton X-100/5% goat serum/PBS and incubated in primary antibodies diluted in 0.2% Triton X-100/5% goat serum/PBS overnight at 4°C. The next day, neurons were washed 3× with PBS and incubated in corresponding secondary antibodies in 0.2% Triton X-100/5% goat serum/PBS for 1 h at room temperature. After washing 4× in PBS, coverslips with cells were briefly submerged into ddH_2_O to remove salts and mounted on Diamond White Glass microscope slides (Globe Scientific, catalog #1,358 W) in a drop of Fluoromount-G (SouthernBiotech, catalog #0100-01) or ProLong Gold Antifade Mountant (Thermo Fisher Scientific, catalog #P36930), if STED microscopy was to be performed. Slides were left to dry in the dark at room temperature for 48 h and then stored at 4°C until imaging.

### Culture electrophysiology

Culture electrophysiology was performed essentially as previously described (Maximov et al., 2007) in whole-cell patch–clamp configuration, holding the cell at −70 mV. Glass pipettes with a resistance of 2–4 MΩ were pulled from borosilicate glass capillaries (World Precision Instruments, catalog #TW150-4) using a PC-10 pipette puller (Narishige Scientific Instrument Laboratory). A MultiClamp 700B amplifier and Digidata 1550A Low-Noise Data Acquisition Digitizer with Clampex 10 data acquisition software (Molecular Devices) were used to monitor synaptic currents, sampled at 4 kHz. For triggering evoked responses, focal square pulse stimuli of 1 ms duration were applied ∼100 µm from the cell soma via a bipolar electrode (FHC) controlled by a Model 2100 Isolated Pulse Stimulator (A-M Systems). Coverslips with neurons were put into an extracellular bath solution that was composed of the following (in mM): 140 NaCl, 5 KCl, 2 MgCl_2_, 10 D-glucose, 10 HEPES (pH 7.4, adjusted with NaOH and 290300 mOsm). The solution also contained 2 mM or 0.5–0.75 mM CaCl_2_ for recording excitatory or inhibitory events, respectively. To pharmacologically isolate excitatory postsynaptic currents, we added the GABA_A_ receptor blocker picrotoxin (50 µM) to the extracellular bath solution, while inhibitory postsynaptic currents were observed in the presence of AP5 (50 µM) and CNQX (10 µM) to block NMDA or AMPA receptors, respectively. To block action potentials during the recording of spontaneous miniature excitatory/inhibitory postsynaptic currents (mEPSCs/mIPSCs) 1 µM tetrodotoxin (TTX) was added to the bath solution. Drugs mentioned above were obtained from Tocris Bioscience. The pipette solution for excitatory recordings contained the following (in mM): 135 Cs-methanesulfonate, 8 NaCl, 10 HEPES, 0.3 EGTA, 0.3 Na2GTP, 2 MgATP, 7 phosphocreatine, 0.1 spermine, and 10 QX-314 (Tocris Bioscience, catalog #1014; pH 7.3, adjusted with CsOH and 306 Osm). For inhibitory recordings, the pipette solution was composed of the following (in mM): 135 CsCl, 10 HEPES, 1 EGTA, 4 MgATP, 0.4 Na2GTP, 7 phosphocreatine, 1 spermine, and 10 QX-314 (Tocris Bioscience, catalog #1014; pH 7.3, adjusted with CsOH and 283 mOsm). Traces were analyzed while blinded to the experimental conditions using Clampfit 10 software (Molecular Devices). mEPSCs/mIPSCs were automatically detected in a template-based search and visually inspected for inclusion or rejection.

### Sparse lentiviral infections and neonatal injections

P0 Lphn1 cKO/Ai14 mutant mice (gender unknown) were anesthetized for 5 min on ice. Concentrated lentivirus was injected with a glass pipette using an infusion pump (Harvard Apparatus). The hippocampal CA1 region was unilaterally targeted using the following coordinates from Lambda: anterior–posterior, + 1.0 mm; medial–lateral, ± 1.1 mm; and dorsal–ventral serial injections at −1.5, −1.3, and −1.1 mm. The flow rate was 1 µl/min, and the injected volume was 0.3 µl. Efficiency and localization of viral expression was confirmed by nuclear GFP expression under a fluorescent microscope.

### Patching acute brain slices

For achieving whole-cell patch–clamp configuration, patch pipettes were pulled from borosilicate glass capillary tubes (TW150-4; World Precision Instruments) using a PC-10 pipette puller (Narishige Scientific Instrument Laboratory). Pipette resistance with intracellular solution ranged from 3 to 5 MΩ. Lentivirus was injected into P0 mice. Infected CA1 pyramidal neurons were analyzed at P18–P30. Transverse hippocampal slices (300 µm) were prepared by cutting in an ice-cold solution containing 228 mM sucrose, 2.5 mM KCl, 1 mM NaH_2_PO_4_, 2 6 mM NaHCO_3_, 0.5 mM CaCl_2_, 7 mM MgSO_4_, and 11 mM D-glucose saturated with 95% O_2_/5% CO_2_. Slices were then transferred to a holding chamber containing ACSF: 11 mM NaCl, 2.5 mM KCl, 1 mM NaH_2_PO_4_, 26 mM NaHCO_3_, 2.5 mM CaCl_2_, 1.3 mM MgSO_4_-7H_2_O, 11 mM D-glucose, and ∼292 mOsm. Slices were recovered at 32°C for 30 min in a water bath and then at room temperature for at least 1 h before recording. Slices were then transferred to a recording chamber where they were continuously perfused with oxygenated ACSF maintained at 32°C. A whole-cell pipette solution was used containing 146 mM CsCl, 10 mM HEPES, 0.25 mM EGTA, 2 mM MgATP, 0.3 mM Na_2_GTP, 0.1 mM spermine, and 7 mM phoshocreatine. The pH of the solution was adjusted to 7.25–7.3 with 1 M CsOH, and osmolarity was between 294 and 298 mOsm.

### IHC of cryosections

During whole-cell patch–clamp configuration, cells were filled for 10–15 min with 2 mg/ml biocytin (Sigma-Aldrich #501787307) in whole patch solution (see above, Patching acute brain cells). The recording pipette was slowly removed from the cell after recording was completed. Acute slices were washed briefly with PBS to remove excess ACSF solution and then transferred to a 4% PFA (Electron Microscopy Sciences, catalog #15714) in PBS solution at 4°C overnight. Slices were then washed with PBS 5× for 5 min. The permeabilization solution (0.3% Triton X-100 in PBS) was applied for 30 min at room temperature. Slices were then placed in blocking solution containing 5% normal goat serum (Jackson ImmunoResearch Laboratories, 005000121) + 0.1% Triton X-100 for 1 h at room temperature. Avidin-Alexa Fluor 488 (Invitrogen, catalog #S32354; 1:1,000) in blocking solution was added to slices for incubation at room temperature for 1.5 h. Slices were washed 1× for 5 min with DAPI (Sigma-Aldrich, catalog #10236276001; 1:1,000) in PBS, 3× for 5 min with PBS, and then mounted on UltraClear microscope slides (Denville Scientific, catalog #M1021) using 10 µl ProLong Gold Antifade reagent (Invitrogen, catalog #P36930).

### Imaging

Standard confocal imaging was performed on a Nikon A1 Eclipse Ti confocal microscope with 10× (air)/20× (air)/60× (oil) objectives (Apo, NA 1.4) and NIS-Elements AR acquisition software. Experiments were performed with constant laser intensities and acquisition settings for all conditions. Optimal Nyquist *xy*-resolution and *Z*-stacks with 0.3 µm/1 µm spacing were used for visualizing synaptic puncta or spines/neuronal morphology, respectively. Confocal image analysis was mostly conducted using NIS-Elements and ImageJ/Fiji. Synaptic puncta and spines on cultured neurons were counted on multiple secondary and tertiary dendrites, and these values were averaged per neuron. Imaris software was used for automatic tracing of axons and dendrites. 2D-STED microscopy was performed on a Nikon Ti2-E microscope stand equipped with a CFI Plan Apo Lambda 100× (oil) objective (NA 1.45) and a STEDYCON confocal and STED module (Abberior). Pulsed excitation lasers were of 488 nm/595 nm/640 nm wavelength, and a 775 nm pulsed STED laser was used for depletion, allowing for 60 nm resolution in all channels. Photons were detected using time-gated avalanche photodiodes with 15-line accumulations and a pixel size of 30 × 30 nm. Raw images were processed in Huygens Essentials (Scientific Volume Imaging) using Express Deconvolution and exported as ICS files for further analysis in NIS-Elements and ImageJ/Fiji. Synapses were defined as the colocalization of pre- and postsynaptic structures. Any myc-positive object colocalizing with a synapse that was smaller than the actual synaptic junction was considered a synaptic nanocluster of Lphn1. GAD67/NeuN-stained neurons and Myc-stained brain slices were imaged using a VS120 slide scanner (Olympus) at 20× (air) magnification, exported as 8 bit OME files, and analyzed in NIS-Elements and ImageJ/Fiji. Dendritic spine images in brain slices were acquired using a Nikon A1r resonant scanning Eclipse Ti2 HD25 confocal microscope with 10× (Nikon MRD00105, CFI60 Plan Apochromat Lambda, NA 0.45), 20× (Nikon MRD00205, CFI60 Plan Apochromat Lambda, NA 0.75), and 60× (Nikon MRD01605, CFI60 Plan Apochromat Lambda, NA 1.4) objectives, operated by the NIS-Elements AR v4.5 acquisition software. For biocytin analysis, images were collected with the following resolution: 20×, 0.86 µM/pixel; 60×, 0.14 µM/pixel. Dendrites from the stratum oriens, stratum radiatum, and stratum lacunosum-moleculare regions were analyzed separately. Multiple secondary/tertiary dendrites were analyzed per neuron, and the resulting values were averaged. Image analysis was conducted using NIS-Elements and Adobe Photoshop for figure purposes. Brightness was adjusted uniformly across all pixels for a given experiment for figure visualization purposes.

### Statistical analyses

The experimenters were blinded to the examined conditions while carrying out experiments and analyzing data. Bar graphs indicate means ± SEM; the distribution of biological replicates (means of culture/individual mice) and—in the case of culture experiments—values for individual synapses/neurons are depicted on the right of each bar as large or small dots, respectively. Statistics were mostly done using a paired *t* test on culture means; for electrophysiological recordings and in vivo experiments, a Mann–Whitney test on values of individual neurons or mice was performed. *P* values >0.1 are not shown in graphs, *p* = 0.05–0.1 is reported numerically: *, *p* < 0.05; **, *p* < 0.01; and ***, *p* < 0.001.

## Results

### Generation and validation of Lphn1 conditional knock-in/cKO mice

**Figure 1.**
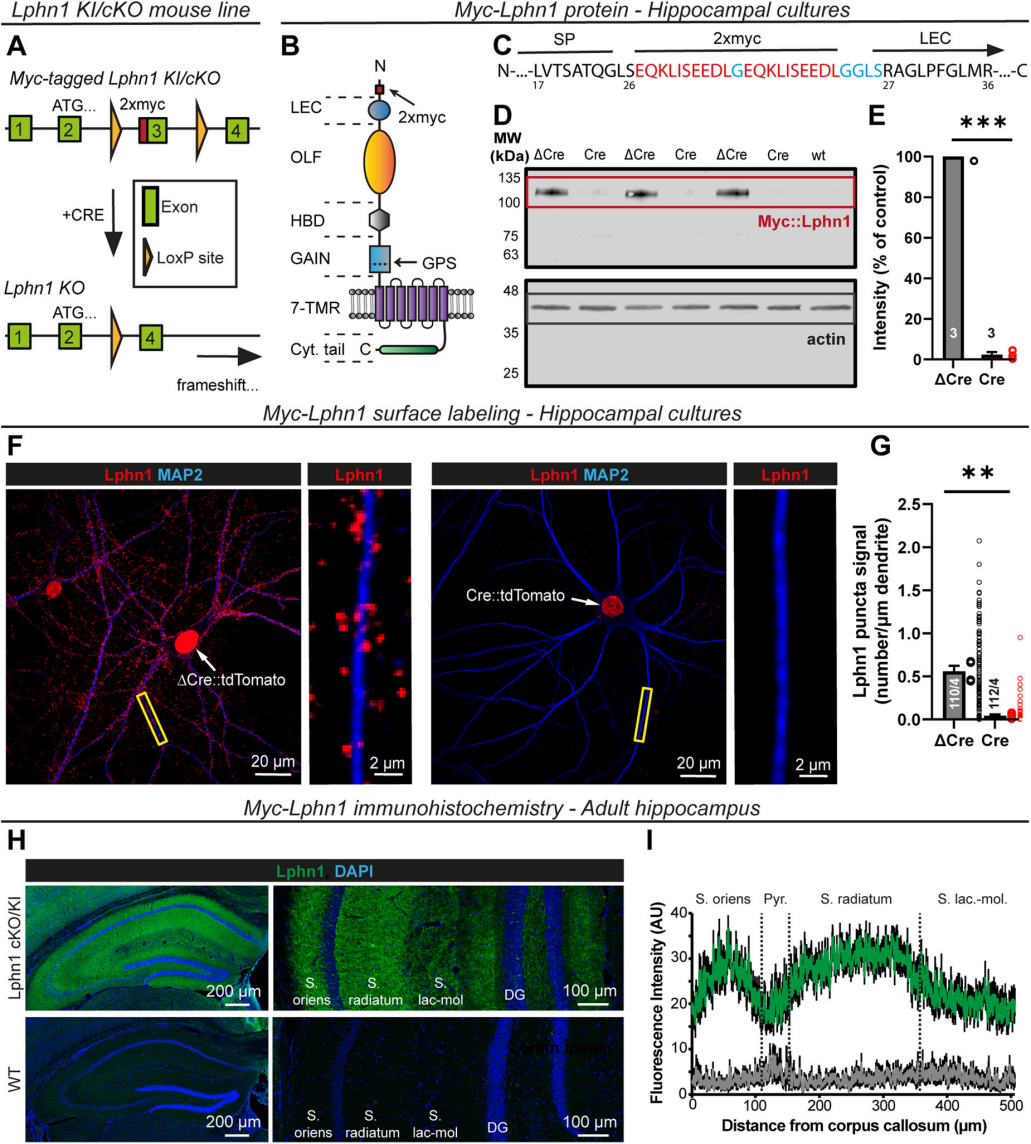
Generation of Lphn1 cKO mice in which endogenous Lphn1 carries an N-terminal 2xmyc tag. ***A***, Design of Lphn1 cKO mice. A 2xmyc tag sequence was introduced into the third exon (start, 84,645,449; end, 84,645,662; Transcript Adgrl1-207-ENSMUST00000141158.8 in GRCm39) of the Lphn1 gene (Adgrl1, ENSMUSG00000013033), and the exon was flanked by loxP sites using homologous recombination in ES cells. ***B***, Model of the resulting myc-tagged Lphn1 protein [adapted from Sando et al. (2019)]. The 2xmyc tag was inserted on the N terminus, just after the signal peptide and before the lectin-like domain (LEC). Other domains of the Lphn1 N terminus are the olfactomedin-like domain (OLF), hormone-binding domain (HBD), and GAIN domain, which contains the site of autoproteolytic cleavage (GPS) typical for aGPCRs. These are followed by a seven-transmembrane receptor domain (7TMR) and a long cytoplasmic tail (Cyt. tail). ***C***, Amino acid sequence of the modified Lphn1 N terminus. The 2xmyc tag is colored in red, linker amino acids in blue, and the flanking endogenous amino acids in black. The location of the signal peptide (SP), 2xmyc tag, and the start of the lectin-like domain (LEC) are given above the sequence, and numbers of amino acid positions of the unmodified Lphn1 protein (Uniprot: Q80TR1) are given below. ***D***, IB validation of Lphn1 cKO mice. Three independent primary hippocampal cultures from Lphn1 cKO mice were infected with lentiviruses encoding active (Cre) or enzymatically inactive recombinases (ΔCre) that contained a nuclear localization signal and were fused to tdTomato. In addition, hippocampal cells from a single wild-type (wt) culture were analyzed. Blots were stained for the knocked-in myc-tag (top) or actin as a loading control (bottom). Note that owing to GAIN domain-mediated autocleavage, the myc-tag labels the N-terminal Lphn1 fragment of ∼115 kDa instead of uncleaved Lphn1 at ∼210 kDa. Full blot images are shown in Extended Data Figure 1-1. ***E***, Quantification of the myc-Lphn1 signal in ***D***. Signals after subtraction of the wt background were normalized to actin and the ΔCre condition (means ± SEMs; *n* = 3 independent cultures; statistics were performed using a paired *t* test, with ***, *p* < 0.001). ***F***, Surface labeling of myc-tagged Lphn1 in primary hippocampal cultures from Lphn1 cKO mice, infected with lentiviruses as described in ***D***. After myc-staining and permeabilization, neurons were additionally stained for MAP2 to visualize dendrites. Myc-Lphn1 staining shows punctate structures concentrated at dendrites that are largely absent in the Cre condition. The nuclear red fluorescence is derived from the expressed Cre- or ΔCre-tdTomato fusion proteins. ***G***, Quantification of myc-Lphn1 puncta per µm of dendrite as shown in ***F*** (means ± SEMs; large dots, mean values for independent cultures; small dots, individual neuronal values; *n* = 110 neurons/4 independent cultures; statistics were performed using a paired *t* test, with **, *p* < 0.01). ***H***, IHC of cryosections from adult Lphn1 cKO and wt control mice stained for the myc epitope, showing that myc-Lphn1 is expressed in all layers of the murine hippocampus. ***I***, Quantification of the myc-Lphn1 fluorescence intensity of stained brain slices from ***H*** as a function of the CA1 region hippocampal layer [*n *= 5 (control) or 7 (Lphn1 cKO); green, Lphn1 cKO mice carrying a myc epitope tag; gray, wt control].

Earlier studies of Lphn2 and Lphn3 were facilitated by the availability of conditional knock-in/cKO mice in which endogenous Lphn2 or Lphn3 was tagged with an epitope for immunocyto-/immunohistochemical localization but could be deleted by the expression of Cre recombinase (Anderson et al., 2017; Sando et al., 2019). Although constitutive Lphn1 KO mice were described (Tobaben et al., 2002; Vitobello et al., 2022), breeding Lphn1 KO mice is challenging because of a major behavioral phenotype (Tobaben et al., 2002; Vitobello et al., 2022). Moreover, no highly specific Lphn1 antibodies are available. To overcome these challenges, we generated new Lphn1 conditional knock-in/cKO mice in which endogenous Lphn1 is tagged at the N-terminus with a 2xmyc epitope but can be deleted with Cre recombinase (Fig. 1*A*–*C*).

10.1523/JNEUROSCI.1978-23.2024.f1-1 Figure 1-1 **Full immunoblot images for the Lphn1 myc-tag epitope knockin/conditional knockout validation.** (A, B) Full images of immunoblots shown in Fig. 1D. myc-tag (A) and actin staining (B) were done with distinct secondary antibodies on the same membrane. Staining for the myc tag shows a specific band at ∼115  kDa in the ΔCre condition but not in the Cre condition and the wild-type (wt) control, corresponding to the cleaved Lphn1 N terminus. Staining for actin (∼40  kDa) shows equal amount of protein has been loaded in all conditions. Unspecific bands are observed with the myc-tag immunoblotting that are not changed by the expression of Cre recombinase and are also present in wild-type controls. Download Figure 1-1, TIF file.

Lphn1 cKO mice were viable and fertile with no apparent behavioral abnormality. To validate the Lphn1 myc-tagging and conditional Lphn1 deletion, we cultured primary hippocampal cells from newborn mice and infected them with lentiviruses expressing inactive mutant ΔCre recombinase (as a control) or active Cre recombinase (test) under control of the neuronal synapsin-1 promoter. Both active Cre and mutant inactive ΔCre recombinase were expressed as tdTomato fusion proteins that contain a nuclear localization signal which translocates the Cre and ΔCre proteins into the nucleus. Upon expression of Cre but not ΔCre, the floxed exon in Lphn1 cKO neurons is recombined, leading to a frameshift in the mRNA that abolishes Lphn1 protein synthesis. To test the efficiency of Cre recombination, we analyzed total protein from infected cultures by quantitative immunoblot (IB) (Fig. 1*D*, Extended Data Fig. 1-1). Whereas ΔCre virus-infected cultures exhibited a ∼115 kDa band corresponding to the Lphn1 N-terminal fragment that results from physiological autocleavage by Lphn1's GAIN domain (Araç et al., 2012), cultures infected with Cre viruses showed *a* > 97% reduction of Lphn1 protein expression (Fig. 1*D*,*E*). Thus, Cre expression abolished Lphn1 expression in the cultured cells.

We next performed surface labeling of the myc-Lphn1 protein in the same hippocampal cultures (Fig. 1*F*). We observed punctate myc signals along dendrites in the ΔCre condition that were largely absent from neurons in the Cre condition (Fig. 1*F*,*G*), consistent with the Lphn1 protein deletion. Furthermore, when we stained cryosections of the brains from adult mice for myc-Lphn1 protein, we found that myc-Lphn1 was uniformly distributed among the various strata of the hippocampus (Fig. 1*H*,*I*), indicating that the Lphn1 distribution differs from that of its paralogs Lphn2 and Lphn3 that are highly enriched in the S. lacunosum-moleculare (Lphn2) or the S. oriens and S. radiatum (Lphn3; Anderson et al., 2017; Sando et al., 2019).

### Lphn1 forms nanoclusters in both excitatory and inhibitory synapses

**Figure 2.**
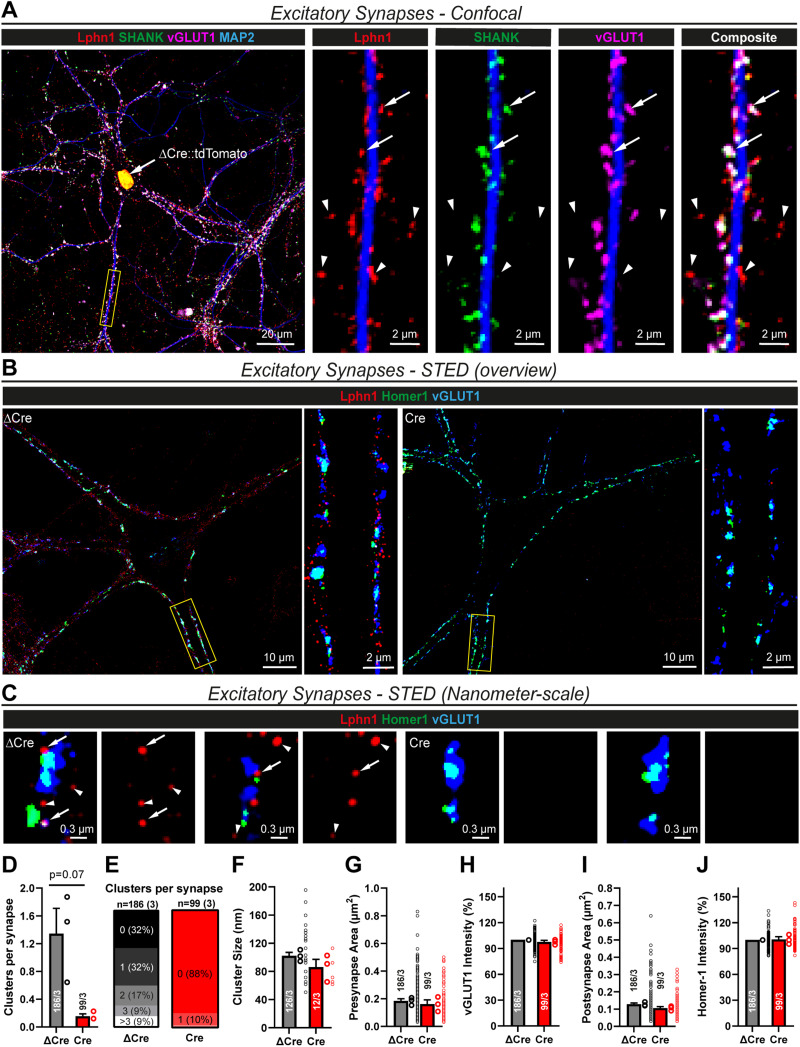
Lphn1 forms nanoclusters in excitatory synapses. ***A***, Colocalization of myc-Lphn1 with excitatory synapse markers using standard diffraction-limited confocal microscopy. After surface labeling for myc-Lphn1, Lphn1 cKO hippocampal cultures were permeabilized and stained for presynaptic (vGLUT1) and postsynaptic (SHANK) markers. Lphn1 is expressed in excitatory synapses (arrows) but also shows Lphn1 localization to puncta that are not stained for excitatory synapse markers (arrow heads). ***B***, ***C***, STED microscopy of Lphn1 cKO hippocampal cultures reveals nanoclusters of Lphn1 in excitatory synapses. Cultures were infected with lentiviruses encoding active (Cre) or mutant inactive Cre recombinase (ΔCre), surface labeled for myc-Lphn1, permeabilized, and stained for presynaptic (vGLUT1) and postsynaptic (Homer1) markers of excitatory synapses. Subsets of Lphn1 nanoclusters colocalize (arrows) or do not colocalize with excitatory synapse markers (arrowheads). Almost no nanoclusters can be found on neurons expressing Cre recombinase. Images are shown at different scales: an overview with zoom-in on a dendrite (***B***) and single synapses (***C***). ***D–J***, Quantification of Lphn1 nanoclusters by STED microscopy as shown in ***B*** and ***C***: mean number of Lphn1 nanoclusters per synapse (***D***); distribution of Lphn1 nanocluster numbers among synapses (***E***); nanocluster sizes (***F***), pre- and postsynapse areas (***G, I***), and vGLUT1- and Homer1-staining intensities normalized to ΔCre (***H, J***). All summary graphs are means ± SEMs. Numbers in bars show the number of synapses/cultures analyzed. Statistics were done using paired *t* tests, but no significant differences were detected.

**Figure 3.**
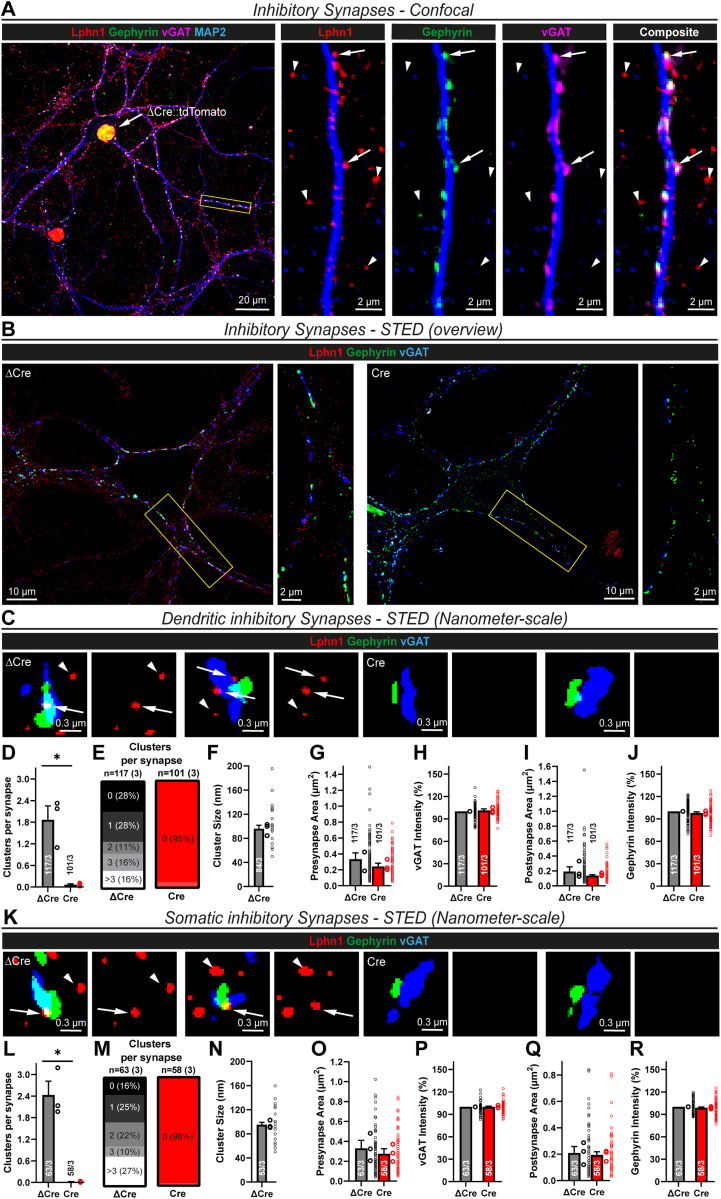
Lphn1 forms nanoclusters in dendritic and somatic inhibitory synapses similar to excitatory synapses. ***A***, Colocalization of myc-Lphn1 with inhibitory synapse markers using standard diffraction-limited confocal microscopy. After surface labeling for myc-Lphn1, Lphn1 cKO hippocampal cultures were permeabilized and stained for presynaptic (vGAT) and postsynaptic (Gephyrin) markers. Lphn1 is expressed in inhibitory synapses (arrows) but also present in puncta that do not stain for inhibitory synapse markers and could represent excitatory synapses (arrowheads). ***B***, STED microscopy of Lphn1 cKO hippocampal cultures reveals nanoclusters of Lphn1 in inhibitory synapses. Cultures were infected with lentiviruses encoding Cre recombinase or functionally deficient ΔCre recombinase, surface labeled for myc-Lphn1, and then permeabilized and stained for presynaptic (vGAT) and postsynaptic (Gephyrin) markers of inhibitory synapses. Almost no nanoclusters can be found on neurons expressing Cre recombinase. ***C***, Single dendritic synapses from ***B*** shown at a smaller scale. Subsets of Lphn1 nanoclusters colocalize with inhibitory synapse markers (arrows), while others do not and are possibly from excitatory synapses (arrowheads). ***D–J***, Quantification of Lphn1 nanoclusters in dendritic inhibitory synapses as shown in ***B*** and ***C***: mean amount of Lphn1 nanoclusters per synapse in ***D*** and number of synapses with no or a certain number of nanoclusters in ***E***, cluster diameter in ***F***, pre- and postsynapse areas in ***G*** and ***I***, and staining intensities of pre- and postsynaptic markers normalized to ΔCre in ***H*** and ***J***. ***K***, Single somatic synapses from ***B*** shown at a smaller scale. Lphn1 nanoclusters colocalize with inhibitory synapse markers (arrows), while others are possibly extrasynaptic (arrowheads). ***L–R***, Quantification of Lphn1 nanoclusters in somatic inhibitory synapses as shown in ***B*** and ***K***: mean amount of Lphn1 nanoclusters per synapse in ***L*** and number of synapses with no or a certain number of nanoclusters in ***M***, cluster diameter in ***N***, pre- and postsynapse areas in ***O*** and ***Q***, and staining intensities of pre- and postsynaptic markers normalized to ΔCre in ***P*** and ***R***. All summary graphs (***D–J*** and ***L–R***) are means ± SEMs. Numbers in bars show number of synapses/cultures analyzed. Statistics were done using paired *t* tests (*, *p* < 0.05).

Lphn2 and Lphn3 are essential for excitatory but not inhibitory synapse formation and are localized to excitatory synapses, although their possible presence in inhibitory synapses has not been investigated (Anderson et al., 2017; Sando et al., 2019). We therefore examined whether Lphn1 localizes to excitatory and/or inhibitory synapses. As an initial approach, we surface-labeled myc-tagged Lphn1 in primary hippocampal cultures from the Lphn1 knock-in/cKO mice, followed by detergent permeabilization of the cells and staining for excitatory and inhibitory synaptic markers (Figs. 2, 3). We observed prominent colabeling of myc-Lphn1 with synaptic markers but found that, surprisingly, Lphn1 colocalized with both excitatory (Fig. 2*A*) and inhibitory synaptic markers (Fig. 3*A*) in the cultured neurons.

Since conventional confocal microscopy is diffraction-limited and poorly resolves individual synapses, we aimed to confirm the presence of Lphn1 in both excitatory and inhibitory synapses using STED microscopy. To ensure that the Lphn1 signal we observe is specific, we examined both ΔCre (Lphn1-expressing) and Cre (Lphn1-deleted) hippocampal cultures and stained them as described above. Intriguingly, we found that Lphn1 forms nanoclusters in excitatory synapses (Fig. 2*B*,*C*) as previously shown for other synaptic adhesion proteins, for example, for the Lphn ligand Teneurin-3 (Zhang et al., 2022). The majority of synapses in the ΔCre condition displayed one to three nanoclusters, while the nanoclusters were absent in ∼90% of synapses in the Cre condition (Fig. 2*D*,*E*). The nanoclusters had a mean diameter of ∼90–100 nm (Fig. 2*F*), similar to the previously published dimensions of teneurin nanoclusters (Zhang et al., 2022). The deletion of Lphn1 did not significantly influence the area or staining intensity of pre- or postsynaptic specializations (Fig. 2*G*–*J*), consistent with largely intact excitatory synapses after the Lphn1 deletion.

We next investigated inhibitory synapses. As suggested by the conventional confocal microscopy, Lphn1 nanoclusters were prominently observed in inhibitory synapses, both on dendrites (Fig. 3*B*,*C*) and the cell soma (Fig. 3*B*,*K*). As a negative control, almost no clusters were found in inhibitory synapses in Cre-expressing neurons lacking Lphn1 (Fig. 3*D*,*L*). Interestingly, in the ΔCre condition, the number of nanoclusters per synapse was on average higher in somatic (mean, ∼2.4 nanoclusters, Fig. 3*L,M*) than in dendritic inhibitory synapses (mean, ∼1.8 nanoclusters, Fig. 3*D*,*E*), which in turn was higher than the average number of nanoclusters in excitatory synapses (mean, ∼1.3 nanoclusters, Fig. 2*D*,*E*). However, the nanocluster size of ∼90–100 nm was similar in all synapse types (Figs. 2*F*, 3*F*,*N*). Pre- and postsynaptic areas had a small, statistically insignificant trend to being smaller in the Cre condition, while the synapse-marker staining intensities were unchanged between ΔCre- and Cre-expressing neurons (Fig. 3*G*–*J*, *O*–*R*).

These data suggest that Lphn1 is localized to the majority of both excitatory and inhibitory synapses in cultured hippocampal neurons and that it assembles into similar nanoclusters in these synapses, although it is on average more abundant in inhibitory synapses.

### Lphn1 is not required for neuronal morphogenesis

**Figure 4.**
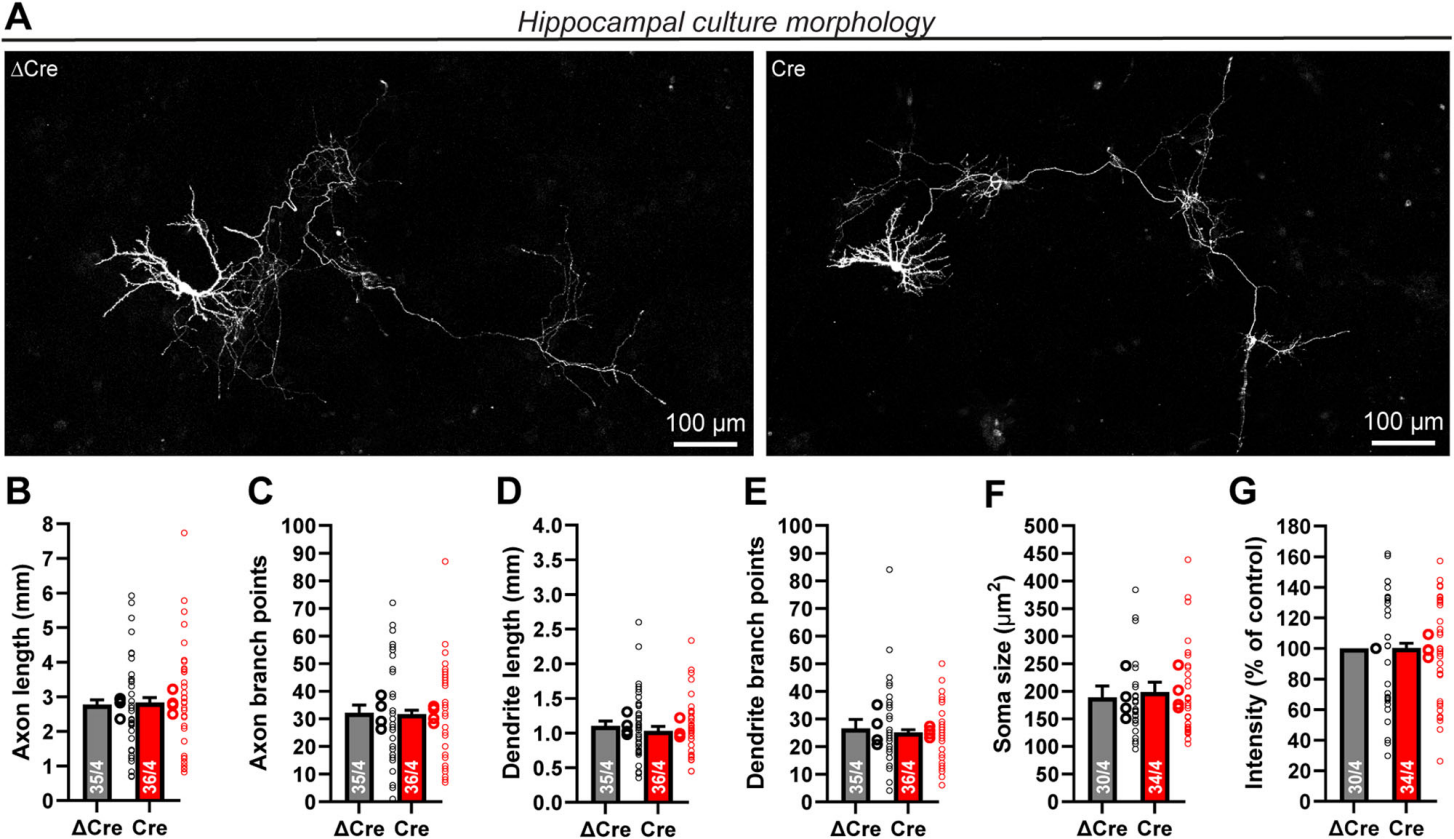
Lphn1 is not required for dendritic or axonal arborization. ***A***, Sample images of sparsely labeled cultured hippocampal neurons that had been infected with lentiviruses encoding active (Cre) or mutant inactive (ΔCre) Cre recombinase. Sparse transfection of a CMV promoter-driven EGFP allows tracing of dendrites and axons of individual neurons. Costaining with MAP2 (data not shown) was used to distinguish axons (MAP2 negative) and dendrites (MAP2 positive). White false color was used for the GFP signal for better contrast. ***B–G***, The Lphn1 deletion does not cause statistically significant changes in total axon length (***B***), axon branch points (***C***), total dendrite length (***D***), dendrite branch points (***E***), or soma size (***F***). Soma intensity was measured to show in ***G*** that similar amounts of GFP are expressed in both conditions and are displayed normalized to the ΔCre condition. All summary graphs (***B–G***) are means ± SEMs. Numbers in bars show number of neurons/cultures analyzed. Statistics were done using paired *t* tests; no significant differences were noted.

Earlier analyses of constitutive Lphn1 KO neurons suggested that Lphn1 interactions with teneurins promotes axonal outgrowth (Vysokov et al., 2018), whereas Lphn3 was proposed to mediate an axon-repellant signal (Pederick et al., 2021). Since functions in dendritic or axonal outgrowth could confound analysis of a role for Lphn1 in synapse assembly, we examined neuronal morphology in developing hippocampal neurons cultured from Lphn1 cKO mice. We lentivirally expressed Cre or ΔCre in the neurons and additionally sparsely transfected them with an EGFP expression plasmid, which allowed us to quantify the size and shape of axons and dendrites as a function of the Lphn1 deletion (Fig. 4*A*–*G*). However, we detected no significant morphological changes caused by the Lphn1 deletion. In particular, the total axon length (Fig. 4*B*), number of axon branch points (Fig. 4*C*), total dendrite length (Fig. 4*D*), number of dendrite branch points (Fig. 4*E*), and soma size (Fig. 4*F*) were not altered in Lphn1-deficient neurons compared with control neurons analyzed in parallel. The GFP fluorescence intensity as a measure of transfection efficiency was also unchanged between groups (Fig. 4*G*). Thus, in culture, the Lphn1 deletion had no effect on axonal or dendritic development.

### Lphn1 is also not required for excitatory synapse assembly

**Figure 5.**
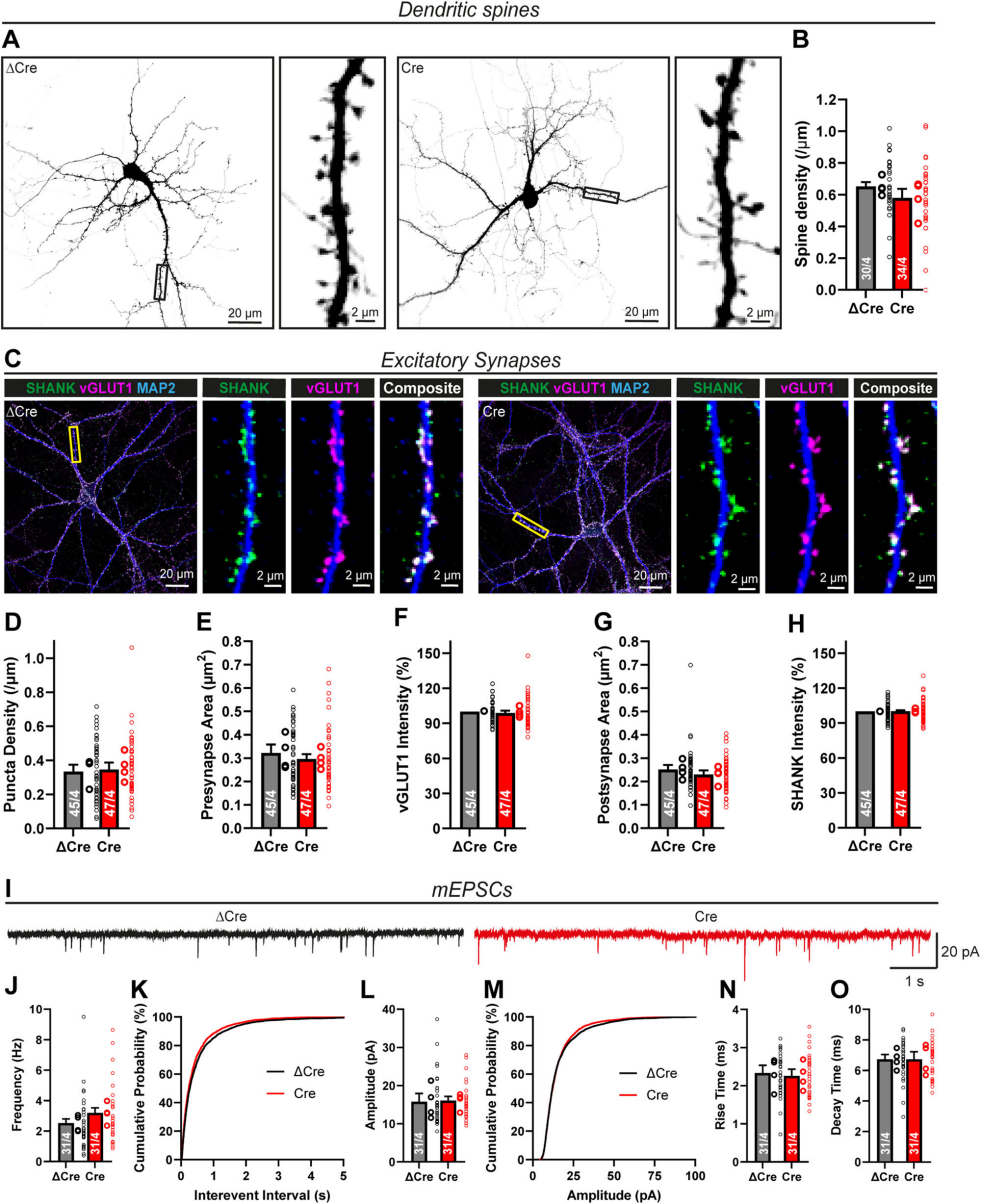
Lphn1 is not required for excitatory synapse formation or spine development. ***A***, ***B***, Spine density analysis in cultured neurons. EGFP-transfected neurons have been imaged at sufficient resolution to count individual spines (***A***). Black false color was used for the GFP signal for better contrast. Only a minor decrease in spine density per micrometer of dendrite was observed, which was not statistically significant (***B***). ***C***, ICC of excitatory synapses in Lphn1-deficient cultures. Lphn1 cKO cultures were infected with lentiviruses encoding Cre recombinase or functionally deficient ΔCre recombinase, permeabilized and stained for presynaptic (vGLUT1) and postsynaptic (SHANK) markers of excitatory synapses as well as MAP2 to visualize dendrites. ***D–H***, Quantification of images as shown in ***C***. No significant changes were observed in any of the following parameters: (***D***) the density of synaptic puncta, defined by colocalized pre- and postsynaptic puncta per µm of dendrite; (***E***, ***G***) area of pre- and postsynaptic puncta; (***F***, ***H***) intensity of the staining of vGLUT1 and SHANK normalized to ΔCre. (***I–O***) Recordings of mEPSCs in the presence of TTX show no significant difference after Lphn1 depletion. Representative traces are shown in ***I***, mEPSC frequency and cumulative plot of the interevent interval in ***J*** and ***K***, mEPSC amplitude and cumulative plot of the amplitude distribution in ***L*** and ***M*** and mEPSC kinetics in ***N*** and ***O***, defined as times of the event to rise from 10 to 90% of its amplitude and conversely to decay from 90 to 10% of its amplitude. Recording parameters and passive cell characteristics for electrophysiological recordings are given in Extended Data Figure 5-1. All summary graphs (***B, D–H*** and ***J–O***) are means ± SEMs. Numbers in bars show number of neurons/cultures analyzed. Statistics were done using paired *t* tests or Mann–Whitney tests (for electrophysiological recordings), but no significant differences were noted.

Previously, deletions of Lphn2 and Lphn3 were shown to decrease spine density and excitatory synapse numbers in hippocampal neurons both in culture and in vivo (Anderson et al., 2017; Sando et al., 2019). We therefore imaged EGFP-transfected–cultured hippocampal neurons expressing or lacking Lphn1 at high resolution to monitor single spines (Fig. 5*A*). Quantifications revealed only a minor trend toward a decreased spine density in Lphn1-deficient neurons, suggesting that different from the Lphn2 and Lphn3 deletions, the Lphn1 deletion does not significantly lower spine numbers (Fig. 5*B*).

10.1523/JNEUROSCI.1978-23.2024.f5-1 Figure 5-1 **Intrinsic membrane properties monitored during electrophysiological recordings are unchanged by the Lphn1 deletion.** Summary graphs show the mean ± SEM access resistance (A), membrane input resistance (B) and membrane capacitance (C). Numbers in bars show the number of neurons/cultures analyzed. Large and small circles next to bars indicate the mean values of each culture and the individual cell values, respectively. Statistics were done using a Mann-Whitney test on individual neuron recordings, but no significant differences have been found. Download Figure 5-1, TIF file.

The lack of a change in spine numbers in Lphn1-deficient neurons suggests that, surprisingly, the Lphn1 deletion may not affect excitatory synapse formation, different from the Lphn2 and Lphn3 deletions (Anderson et al., 2017; Sando et al., 2019). Contrary to this suggestion, however, a previous study showed that cultured hippocampal Lphn1-deficient neurons exhibited a reduction in the staining intensities for presynaptic markers, suggesting a loss of synapses, although no measurements of synapse numbers were performed (Vitobello et al., 2022). To test this question directly, we stained Lphn1-deficient and control neurons by ICC for the vesicular glutamate transporter vGLUT1 and the postsynaptic scaffold protein SHANK (monoclonal antibody recognizing all three isoforms SHANK1–3), which are markers for pre- and postsynaptic excitatory synapses (Naisbitt et al., 1999; Bellocchio et al., 2000; Fig. 5*C*), and measured the synapse density. Consistent with the lack of a change in spine density, the density of synaptic puncta was identical between control and Lphn1-deficient neurons (Fig. 5*D*). The area and staining intensities of pre-and postsynaptic regions were also unchanged (Fig. 5*E*–*H*).

The morphological analyses suggest that the Lphn1 deletion does not affect excitatory synapse numbers in pyramidal neurons. To verify these findings, we performed whole-cell patch–clamp recordings in control (ΔCre) and Lphn1-deficient (Cre) neurons in the presence of TTX, and measured spontaneous mEPSCs (Fig. 5*I*). The mEPSC event frequency was slightly, but not significantly, elevated by the Lphn1 deletion (Fig. 5*J*–*K*), while their amplitude and kinetics were unchanged (Fig. 5*L*–*O*). Recording parameters and passive cell characteristics were similar in both conditions throughout all electrophysiological experiments (Extended Data Fig. 5-1).

**Figure 6.**
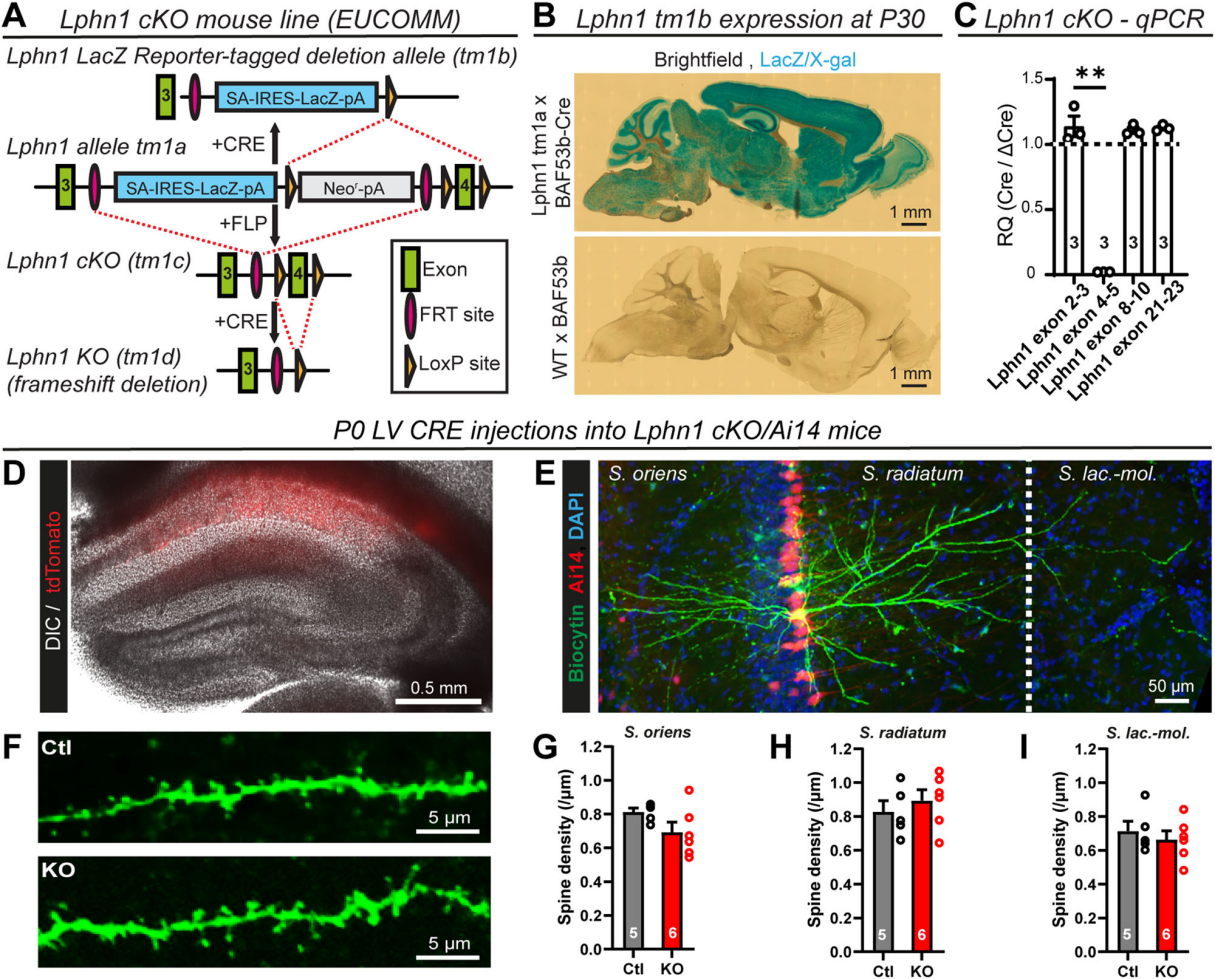
Sparse KO of Lphn1 in vivo does not alter neuronal morphology or spine density. ***A***, A schematic design of Lphn1 cKO mice generated from EUCOMM ES cells (see Materials and Methods, Generation of Lphn1 cKO mice, for details). The original line (tm1a) allows for the introduction of a LacZ reporter in the presence of Cre recombinase. Alternatively, a conditional allele (tm1c), in short Lphn1 cKO, can be generated with FLP recombinase followed by the conditional deletion of the fourth exon (start, 84,649,628; end, 84,649,737; Transcript Adgrl1-207-ENSMUST00000141158.8 in GRCm39) of Lphn1 with Cre. ***B***, LacZ reporter staining in Lphn1 tm1a or wt mice expressing pan-neuronal BAF53B-Cre suggests high neuronal Lphn1 expression throughout the postnatal brain. ***C***, qPCR on mRNA samples from Lphn1 cKO primary hippocampal cultures infected with lentivirus encoding either ubiquitin-driven GFP-deltaCre (control) or GFP-Cre confirms KO of exon 4 in the Cre condition. ***D–I***, Morphological analysis of CA1 pyramidal cell dendrites infected with Cre recombinase lentivirus and noninfected controls in acute slices. ***D***, Overview image of a hippocampus, with Cre-infected cells highlighted by Ai14 (tdTomato) reporter expression in red. ***E***, A 20× image of a biocytin labeled CA1 pyramidal cell and all analyzed regions of interest: stratum oriens, stratum radiatum, and stratum lacunosum-moleculare. ***F***, A representative image of dendritic spines in high magnification. ***G–I***, There was no significant difference in spine density in the stratum oriens (***G***), stratum radiatum (***H***), or stratum lacunosum-moleculare (***I***). All summary graphs are means ± SEMs. Numbers in bars show number of cultures (***C***) or mice (***G–I***) analyzed. Statistics were done using a paired *t* test on culture values or Mann–Whitney test on values of individual mice (**, *p* < 0.01).

The absence of an excitatory synapse phenotype in Lphn1-deficient pyramidal neurons is surprising in view of the robust Lphn1 expression in these synapses, the fact that an excitatory synapse phenotype was reported for a constitutive Lphn1 KO mouse (Vitobello et al., 2022), and the large decreases observed in excitatory synapse and spine numbers in Lphn2- and Lphn3-deficient pyramidal neurons (Anderson et al., 2017; Sando et al., 2019). To independently confirm this observation, we generated a second Lphn1 cKO mouse using ES cells obtained from the EUCOMM consortium (Fig. 6*A*). The initial mice generated in this approach express LacZ from the endogenous Lphn1 gene (*Adgrl1*), enabling an independent assessment of Lphn1 expression. LacZ staining confirmed that Lphn1 is broadly expressed throughout the brain (Fig. 6*B*). We then crossed the EUCOMM mice with a germline Flp recombinase-expressing mouse to generate Lphn1 cKO mice that contain normal Lphn1 levels (Fig. 6*A*). The infection of cultured cKO hippocampal neurons with ubiquitin promoter-driven Cre recombinase resulted in *a* > 97% deletion of the floxed exon compared with a ΔCre-infected control (Fig. 6*C*). We additionally crossed the cKO mice with a Cre-dependent tdTomato reporter mouse line (Ai14) and analyzed the spine density in the Lphn1 cKO/Ai14 mice as a function of the Lphn1 deletion in vivo. Specifically, we stereotactically infected CA1 pyramidal neurons in neonatal Lphn1 cKO mice with Cre-expressing lentiviruses and filled infected Lphn1-deficient and uninfected control cells with biocytin via the patch pipette (Fig. 6*D*,*E*). Subsequent analyses of the spine density as a proxy for synapse density again failed to detect any change in Lphn1-deficient neurons compared with that in control neurons in the S. oriens, radiatum, or lacunosum-moleculare (Fig. 6*F*–*I*). These findings provide further support for the unexpected conclusion that Lphn1 is not required in hippocampal pyramidal neurons for synapse formation.

### Lphn1 is essential for normal assembly of somatic inhibitory synapses

**Figure 7.**
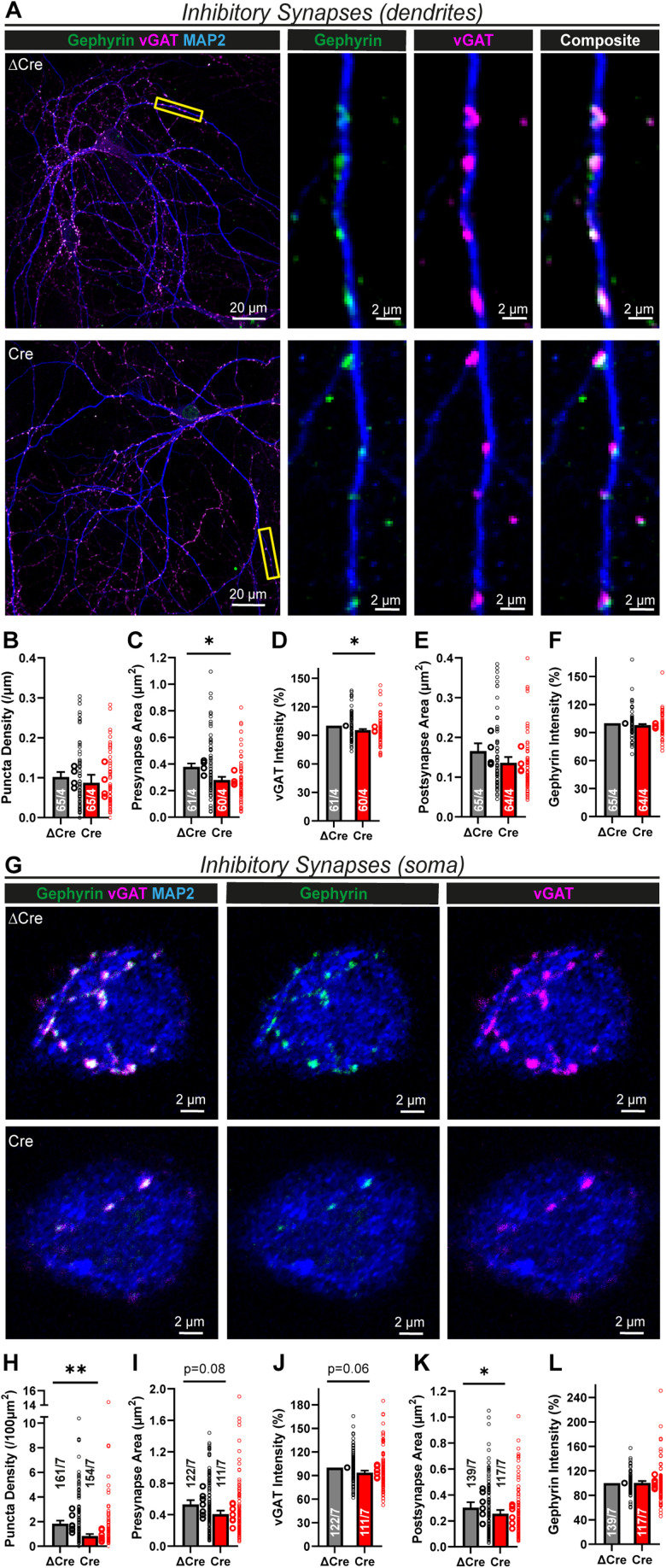
Lphn1 is specifically essential for somatic inhibitory synapse formation. ***A***, ICC of inhibitory synapses in Lphn1-deficient cultures. Lphn1 cKO cultures were infected with lentiviruses encoding Cre recombinase or functionally deficient ΔCre recombinase, permeabilized and stained for presynaptic (vGAT) and postsynaptic (Gephyrin) markers of inhibitory synapses as well as MAP2 to visualize dendrites for synapse density quantifications. ***B–F***, Quantification of images as shown in ***A***. No major changes of the following parameters were observed in the Cre condition: (***B***) the density of synaptic puncta, defined by colocalized pre- and postsynaptic puncta per micrometer of dendrite; (***C, E***) area of pre- and postsynaptic puncta; (***D, F***) intensity of the staining of vGAT and Gephyrin normalized to ΔCre. ***G***, Stained cultures as described in ***A*** were additionally imaged at the cell soma, above the dendritic plane, to assess somatic inhibitory synapses. ***H–L***, Quantification of images as shown in ***G***. Somatic inhibitory synapse density, defined by colocalized pre- and postsynaptic puncta per 100 µm^2^ of soma, was significantly reduced in the Cre condition (***H***), while the area of pre- and postsynaptic puncta and intensity of the staining of vGAT and Gephyrin (normalized to ΔCre) were only marginally reduced (***I–L*)**. All summary graphs (***B–F*** and ***H–L***) are means ± SEMs. Numbers in bars show number of neurons/cultures analyzed. Statistics were done using paired *t* tests (*, *p* < 0.05; **, *p* < 0.01). To rule out a reduction in inhibitory neuron density as a confounding factor for synapse formation, we determined the percentage of GABAergic neurons in cultures infected with ΔCre/Cre virus, using GAD67 staining (Extended Data Fig. 7-1).

Since the expression of Lphn1 in inhibitory synapses seems to be higher than in excitatory synapses (Figs. 2, 3), we next stained cultured Lphn1-deficient and control hippocampal neurons for the vesicular GABA transporter vGAT and the postsynaptic scaffold protein Gephyrin (Langosch et al., 1992; McIntire et al., 1997) (Fig. 7*A*). Dendritic inhibitory synapses lacking Lphn1 were nearly unchanged in density, size, or marker intensity (Fig. 7*B*–*F*), with only a small decrease in size and intensity. When examining somatic inhibitory synapses (Fig. 7*G*), however, we observed a dramatic decrease (∼50–60%) in puncta density (Fig. 7*H*) as well as a minor decrease in pre- and postsynaptic area and staining intensity (Fig. 7*I*–*L*).

10.1523/JNEUROSCI.1978-23.2024.f7-1 Figure 7-1 **Inhibitory interneuron density is unchanged by the Lphn1 deletion.** (A) Representative images of hippocampal neurons cultured from Lphn1 cKO mice. Neurons were infected with lentiviruses expressing active Cre recombinase or inactive mutant ΔCre and immunostained for GAD67 as a marker of inhibitory interneurons and for NeuN as a marker of all neurons. The imaging threshold was set to exclude synaptic GAD67 fluorescence, leaving only somatic fluorescence of interneurons to be analyzed. (B) Quantification of GAD67 + cells (interneurons) as a percentage of NeuN + cells reveals similar interneuron densities in Lphn1-deficient cultures compared to the control condition. (C) GAD67 staining intensity is unchanged between Cre and ΔCre expressing neurons. Multiple coverslips were scanned and pooled for each condition in each culture (n = 3). Summary graphs show means ± SEMs; circles indicate the mean values obtained in a given independent culture. Statistics were done using paired t-tests, but no significant difference has been found. Download Figure 7-1, TIF file.

To ensure that the decrease in inhibitory innervation is not a secondary effect due to lower numbers of inhibitory neurons in cultures infected with Cre virus, we determined the percentage of GABAergic neurons using GAD67 staining (Extended Data Fig. 7-1*A*). In both conditions, we estimated an interneuron density of ∼10% (Extended Data Fig. 7-1*B*). The staining intensity of GAD67 was also not significantly altered (Extended Data Fig. 7-1*C*).

**Figure 8.**
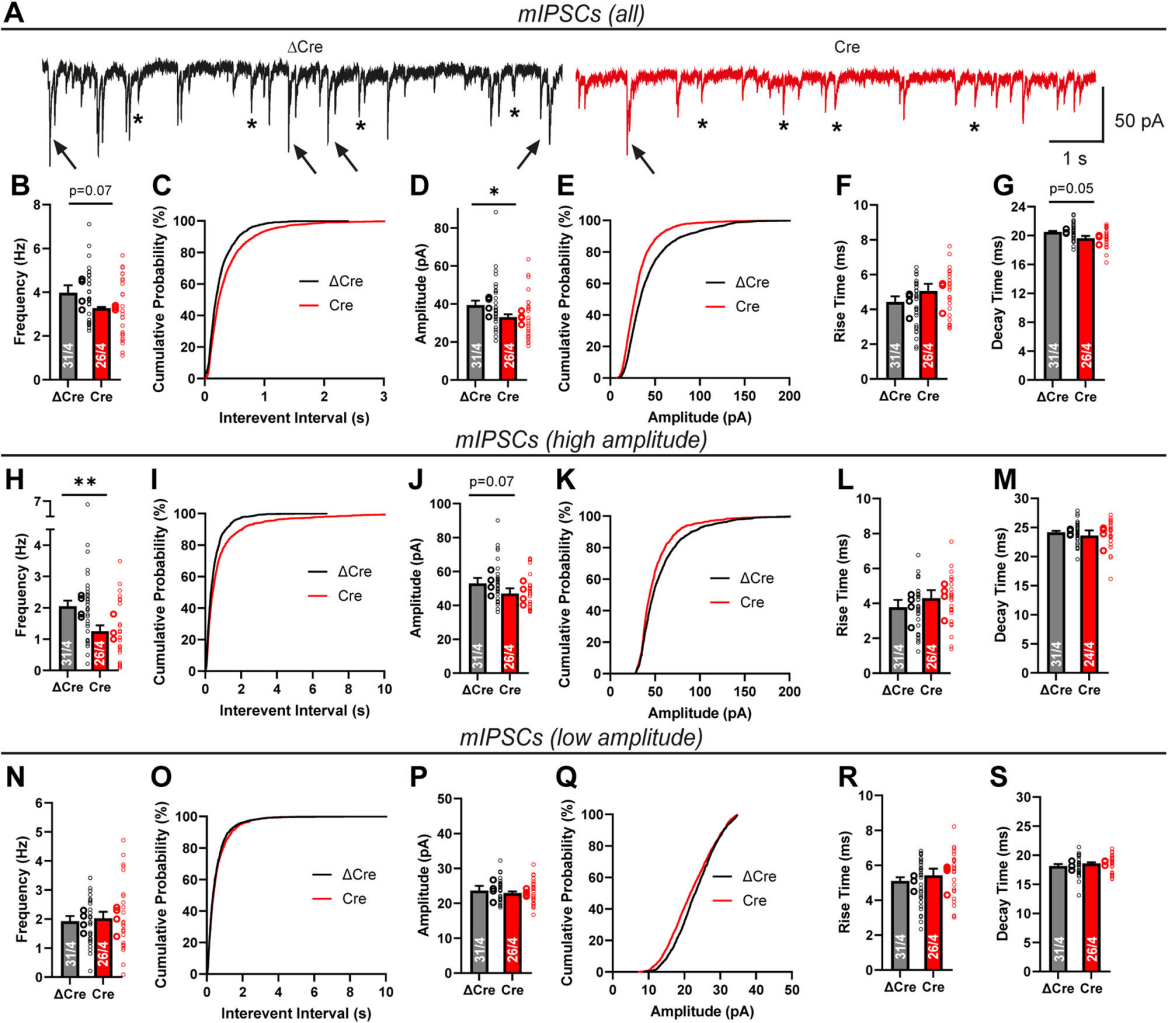
Electrophysiological analyses of cultured hippocampal neurons show that Lphn1 is essential for normal inhibitory synaptic transmission. Lphn1 cKO cultures were infected with lentiviruses encoding Cre recombinase or functionally deficient ΔCre recombinase, and electrophysiological measurements were performed in whole-cell voltage–clamp configuration: (***A–G***) recordings of mIPSCs in the presence of TTX show reduced frequency and amplitude after Lphn1 depletion. Representative traces (***A***) show a specific reduction of high-amplitude mIPSCs (arrows), while low-amplitude mIPSCs are largely unchanged (asterisks). ***B, C***, mIPSC frequency is reduced in the Cre condition, and the cumulative plot of interevent intervals of Cre is shifted toward higher intervals. ***D*, *E***, mIPSC amplitude is significantly reduced, and the cumulative plot of the amplitude distribution is shifted toward smaller amplitudes in the Cre condition. ***F***, ***G***, mIPSC kinetics, defined as times of the event to rise from 10 to 90% of its amplitude and conversely to decay from 90 to 10% of its amplitude, show slightly higher rise times and smaller decay times. ***H–S***, Stratified analysis of high- and low-amplitude mIPSC. For each experiment, an amplitude threshold based on the 50% value of the control condition (ΔCre) was defined (∼32 pA on average) and applied to all analyzed events in both conditions. ***H–M***, Analysis of high-amplitude mIPSCs reveal a strong reduction in frequency after Lphn1 KO (***H***, ***I***), but no significant changes in amplitude, rise time, and decay time (***J–M***). Low-amplitude events do not show any significant differences in frequency, amplitude, or event kinetics (***N–S***). All summary graphs are means ± SEMs. Numbers in bars show number of neurons/cultures analyzed. Statistics were done using Mann–Whitney tests on individual neuron recordings (*, *p* < 0.05; **, *p* < 0.01).

Thus, in contrast to Lphn2 and Lphn3, Lphn1 is not required for excitatory synapse formation but is essential for inhibitory synapse formation, in particular for somatic inhibitory synapses. To verify these findings, we performed whole-cell patch–clamp recordings of mIPSCs in control and Lphn1-deficient hippocampal neurons in the presence of TTX (Fig. 8*A*). When we analyzed total mIPSCs, we observed a minor decrease in mIPSC frequency of ∼20% and a similar decrease in amplitude (Fig. 8*B*–*E*), while the mIPSC kinetics were largely unchanged (Fig. 8*F*,*G*). Interestingly, large-amplitude mIPSCs seemed to be affected more severely than small-amplitude mIPSCs (Fig. 8*A*). mIPSCs originating from the cell soma are thought to exhibit larger amplitudes and faster rise times than mIPSCs generated in more distant dendrites (Wierenga and Wadman, 1999). We therefore stratified mIPSCs into high-amplitude and low-amplitude events using an amplitude threshold, defined as the 50% percentile in the amplitude distribution of the ΔCre condition (average ∼32 pA). Intriguingly, the high-amplitude event frequency was reduced by ∼40–50% in Lphn1 KO neurons (Fig. 8*H,I*), while low-amplitude events were unchanged (Fig. 8*N*,*O*). Amplitudes and kinetics of both subsets were not altered between the Cre and ΔCre conditions (Fig. 8*J*–*M*, *P*–*S*).

**Figure 9.**
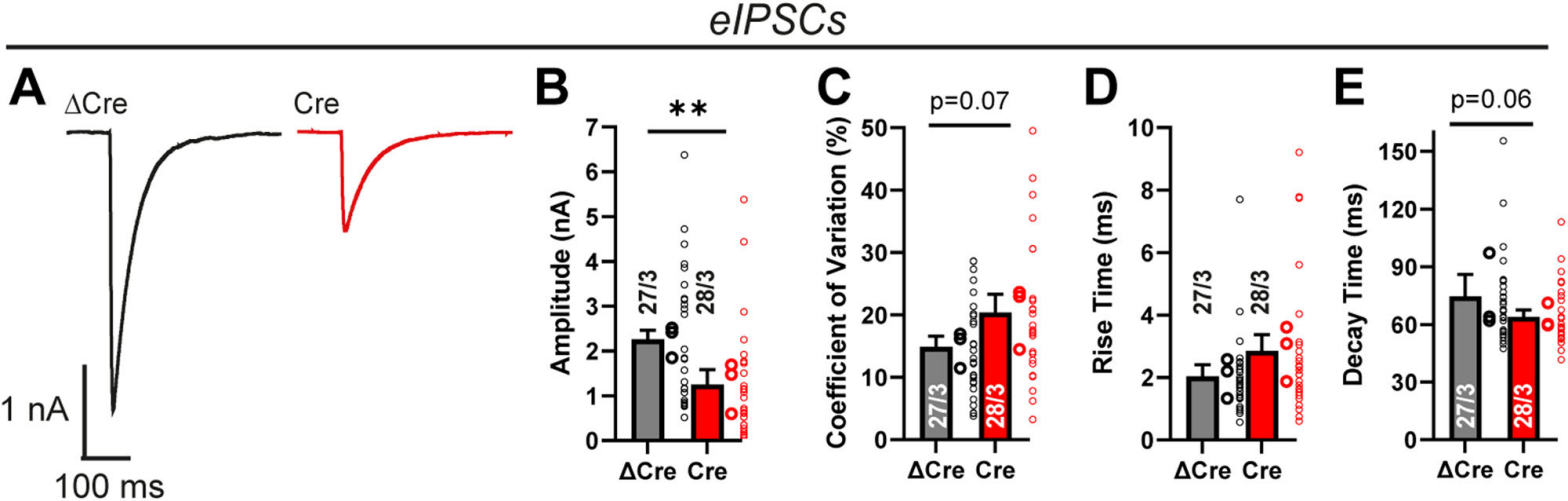
Electrophysiological measurements of evoked responses confirm an essential role of Lphn1 in inhibitory synapses. Recordings of eIPSCs in Lphn1 KO neurons show impaired inhibitory synaptic transmission. Responses of neurons to a 1 ms square pulse stimulus, delivered by a bipolar electrode to the culture coverslip in 100 µm distance from the cell body, were recorded, while excitatory responses were pharmacologically suppressed. Representative traces are shown in ***A***. In the Cre condition, eIPSC amplitude is significantly reduced (***B***), and the coefficient of variation is slightly elevated (***C***). eIPSC kinetics (***D, E***), defined as times of the event to rise from 20 to 80% of its amplitude and conversely to decay from 80 to 20% of its amplitude, were largely unchanged. ***B–E*** show means ± SEMs. Numbers in bars show number of neurons/cultures analyzed. Statistics were done using Mann–Whitney tests on individual neuron recordings (**, *p* < 0.01).

To further validate an inhibitory synapse phenotype induced by the Lphn1 deletion, we next analyzed evoked inhibitory postsynaptic currents (eIPSCs; Fig. 9*A*). We observed a ∼50% decrease in eIPSC amplitude as well as a modest increase in the coefficient of variation (Fig. 9*B*,*C*). These results indicate that the Lphn1 deletion strongly impaired inhibitory synaptic transmission. The effect of the Lphn1 deletion is probably more robust as measured by eIPSCs than as measured by mIPSCs because somatic inhibitory synapses are functionally more potent than dendritic inhibitory synapses since they are closer to the recording electrode. Rise and decay times of eIPSCs were not significantly altered between Cre- and ΔCre-expressing neurons (Fig. 9*D,E*).

## Discussion

In the current study, we generated conditional Lphn1 KO mice that carry a knocked-in N-terminal myc epitope tag to enable us to immunolocalize the Lphn1 protein (Fig. 1*A*–*G*). We show that in the hippocampal CA1 region, Lphn1 is uniformly present throughout the dendritic tree of pyramidal neurons (Fig. 1*H*,*I*), different from Lphn2 and Lphn3 that are enriched in specific strata (Anderson et al., 2017; Sando et al., 2019). Moreover, we find in cultured hippocampal neurons using STED super-resolution microscopy that, unexpectedly, Lphn1 forms nanoclusters in both excitatory and inhibitory synapses (Figs. 2, 3). Intriguingly, we observed that the deletion of Lphn1 caused a significant decrease in the number of somatic but not dendritic inhibitory synapses (Fig. 7). In parallel, we observed reduced inhibitory synaptic strength that most likely was due to the decrease in inhibitory somatic synapses. Specifically, frequencies of high-amplitude mIPSCs but not of low-amplitude mIPSCs (reflecting synaptic events close to or distant from the soma) were significantly decreased (Fig. 8*H*,*N*). Furthermore, eIPSC amplitude was lower in Lphn1-deficient than in control neurons without a significant change in the coefficient of variation (Fig. 9). However, we observed no other major changes in Lphn1-deficient neurons. Neither dendritic nor axonal arborizations were changed (Fig. 4), spine numbers were not decreased, and excitatory synapse numbers or mEPSCs were not significantly changed (Fig. 5). The lack of a change in spines was confirmed in vivo using a second independent Lphn1 cKO mouse line, suggesting that it is not an artifact of one particular mouse line (Fig. 6).

We validated our mouse lines by immunoblot, surface labeling, and qPCR of Lphn1 (Figs. 1*D*–*G*, 6*C*). All methods suggest a highly efficient KO of the targeted exon, which leads to a frameshift and truncation of the resulting protein. However, our qPCR results show persistent expression of mutant mRNAs (Fig. 6*C*). While a likely explanation for this is the escape from non-sense–mediated decay (Embree et al., 2022), another possibility would be alternative splice variants that do not include the exons we targeted. Multiple studies revealed a plethora of alternative splice variants of latrophilins (Sugita et al., 1998; Bjarnadóttir et al., 2007; Boucard et al., 2014; Knierim et al., 2019; Ovando-Zambrano et al., 2019; Wang et al., 2024), which makes the choice of a constitutively included exon essential for complete KO. Since we deleted constitutively included exons (Wang et al., 2024), any alternatively spliced variants lacking these exons that might persist in the KO would be nonfunctional at the synapse.

Our STED super-resolution microscopy data indicate that Lphn1 is one among few postsynaptic adhesion molecules that are in both excitatory and inhibitory synapses, which otherwise share few postsynaptic components (Sheng and Kim, 2011; Harris and Weinberg, 2012; Südhof, 2021). With a diameter of ∼90 nm, the sizes of Lphn1 nanoclusters in excitatory and inhibitory synapses are comparable (Figs. 2*F*, 3*F*,*N*), consistent with similar molecular organization. Moreover, the Lphn1 nanoclusters closely resemble previously observed nanoclusters of presynaptic teneurin-3 (Zhang et al., 2022) and neurexins (Trotter et al., 2019; Lloyd et al., 2023) and of postsynaptic neuroligins (Haas et al., 2018; Han et al., 2022; Nozawa et al., 2022), suggesting that such nanoclusters are a general feature of synaptic adhesion molecules. The detection of synaptic Lphn1 nanoclusters in both excitatory and inhibitory synapses also resolves the previously puzzling observation that teneurins, which are presynaptic latrophilin ligands, are broadly expressed in both excitatory and inhibitory neurons and could thus represent presynaptic interaction partners for postsynaptic Lphn1 in both types of synapses. Furthermore, latrophilins could bind presynaptic neurexins (Boucard et al., 2012), which have been shown to mediate parvalbumin-positive inhibitory synapse formation (Chen et al., 2017).

Our findings raise important new questions. First, are Lphn2 and Lphn3 also present in inhibitory synapses like Lphn1? This seems likely but has not yet been studied at similar levels of resolution as in this study. Second and more puzzlingly, why doesn't the Lphn1 deletion in our experiments produce a major phenotype in excitatory synapses, given that the Lphn2 and Lphn3 deletions cause major impairments in excitatory synapses (Anderson et al., 2017; Sando et al., 2019) and that previous studies on primary hippocampal cultures from constitutive Lphn1 KO mice proposed significant impairments in excitatory synapse function (Vitobello et al., 2022)? A possible explanation for this discrepancy is that the Lphn1 deletions were analyzed on different genetic backgrounds and with conditional versus constitutive deletions. Additionally, Vitobello et al. discuss decreased neuronal viability in constitutively Lphn1-deficient hippocampal cultures, which would lead to reduced synapse density and function. However, our acute Lphn1 deletion does not lead to a significant decrease in inhibitory neuron density (Extended Data Fig. 7-1), making neuron survival an unlikely confounding variable for inhibitory synapse density and function. Furthermore, it seems unlikely that the discrepancy is due to a technical issue in our study or in that of Vitobello et al. (2022), since we have established that our deletion indeed abolishes Lphn1 protein expression as expected from the genetic strategy (Fig. 1*A*–*G*) and the phenotypes we observed in inhibitory synapses (Figs. 7–9) and Vitobello et al. (2022) described in excitatory/inhibitory synapses appear to be very robust. Note, however, that another previous paper on constitutive Lphn1 KO mice using neurotransmitter release from synaptosomes as an assay also did not detect a major change in excitatory synapses, although a synaptic density phenotype would have been missed in that study (Tobaben et al., 2002).

Another difference between the present results and previous analyses of Lphn1 constitutive KO mice is that we did not detect changes in axonal outgrowth (Fig. 4), whereas constitutive Lphn1 KO neurons were found to display axonal growth defects (Vysokov et al., 2018). Functions in neuronal morphogenesis were also reported for the *Caenorhabditis elegans* (*C. elegans*) latrophilin homolog LAT-1 (Matúš et al., 2021). However, only fluorescence intensities of axons were measured in previous analyses, but axon lengths were not tracked (Vysokov et al., 2018). Such methods are easily confounded by variations in culture density or composition. Moreover, it is puzzling that some experiments assign an attractive function of the latrophilin–teneurin interaction in axonal outgrowth (Vysokov et al., 2018), whereas others propose a repellent effect (Pederick et al., 2021). Furthermore, latrophilins were proposed to have an essential role in neuronal migration during brain development (Del Toro et al., 2020), even though major developmental effects on cortical layers by the constitutive Lphn1 deletion were not apparent (Tobaben et al., 2002; Vysokov et al., 2018; Vitobello et al., 2022).

An important question that remains largely unanswered is how latrophilins function on a molecular level to promote excitatory and inhibitory synapse formation. Recent findings suggest G-protein binding of Latrophilin-3 and localized synaptic cAMP signaling to be essential molecular events for excitatory synapse formation (Sando et al., 2021, 2022; Wang et al., 2024). Neuronal isoforms of Latrophilin-1 bind to multiple G-proteins with a preference for Gs (Wang et al., 2024), which could mean that the inhibitory synapses formed by Lphn1 depend on localized Gs-cAMP signaling cascades but might also include other G-proteins. However, analyses of a latrophilin homolog in *C. elegans* found neuronal functions of the molecule that were independent of the 7TM domain and therefore unlikely involve canonical G-protein–mediated signaling (Matúš et al., 2021). It is yet to be determined whether G-protein coupling is a universal necessity for the role of latrophilins in synapse formation or if other unusual modes of aGPCR function could contribute in this context.

In summary, our results expand our view of the synaptic role of latrophilins, suggesting a general function in most synapses independent of their transmitter type. Moreover, our findings uncover an essential function for Lphn1 in somatic inhibitory synapses. Intriguingly, patients carrying pathogenic variants of Lphn1 display behavioral abnormalities including attention-deficit/hyperactivity disorder, autism spectrum disorder, and epilepsy (Vitobello et al., 2022). It is conceivable that a reduction in inhibition and the resulting hyperexcitation could promote such pathologies. However, major questions arose that need to be addressed in the future, such as that of the role of Lphn1 in excitatory synapses. Such a role might be occluded by redundancy, or Lphn1 might be without function in these synapses. Given the differences in results with distinct mouse lines, a more reductionist approach may be needed to deconstruct the contribution of latrophilins in general and Lphn1 in particular to different types of synapses. For example, it is possible that latrophilins function in the same pathway as other aGPCRs, such as BAIs and CELSRs that also have synaptic functions (Bolliger et al., 2011; Najarro et al., 2012; Stephenson et al., 2013; Kakegawa et al., 2015; Sigoillot et al., 2015; Zhu et al., 2015; Martinelli et al., 2016; Thakar et al., 2017; Tu et al., 2018; Wang et al., 2020, 2021; Zhou et al., 2021; Li et al., 2022; Shiu et al., 2022; Aimi et al., 2023; Freitas et al., 2023), and that a more extensive deletion of multiple genes will be necessary to unravel the making of a synapse!

## Data Availability

All numerical data and raw data files have been deposited in the Stanford Data Repository at the following URL: https://doi.org/10.25740/gg154gt4111.

## References

[B1] Aimi T, Matsuda K, Yuzaki M (2023) C1ql1-Bai3 signaling is necessary for climbing fiber synapse formation in mature Purkinje cells in coordination with neuronal activity. Mol Brain 16:61. 10.1186/s13041-023-01048-4 37488606 PMC10367388

[B2] Anderson GR, Maxeiner S, Sando R, Tsetsenis T, Malenka RC, Südhof TC (2017) Postsynaptic adhesion GPCR latrophilin-2 mediates target recognition in entorhinal-hippocampal synapse assembly. J Cell Biol 216:3831–3846. 10.1083/jcb.201703042 28972101 PMC5674891

[B3] Araç D, Boucard AA, Bolliger MF, Nguyen J, Soltis SM, Südhof TC, Brunger AT (2012) A novel evolutionarily conserved domain of cell-adhesion GPCRs mediates autoproteolysis. EMBO J 31:1364–1378. 10.1038/emboj.2012.26 22333914 PMC3321182

[B4] Bailey CH, Kandel ER, Harris KM (2015) Structural components of synaptic plasticity and memory consolidation. Cold Spring Harb Perspect Biol 7:a021758. 10.1101/cshperspect.a021758 26134321 PMC4484970

[B5] Basu J, Siegelbaum SA (2015) The corticohippocampal circuit, synaptic plasticity, and memory. Cold Spring Harb Perspect Biol 7:a021733. 10.1101/cshperspect.a021733 26525152 PMC4632668

[B6] Bellocchio EE, Reimer RJ, Fremeau RT, Edwards RH (2000) Uptake of glutamate into synaptic vesicles by an inorganic phosphate transporter. Science 289:957–960. 10.1126/science.289.5481.957 10938000

[B7] Bjarnadóttir TK, Geirardsdóttir K, Ingemansson M, Mirza MAI, Fredriksson R, Schiöth HB (2007) Identification of novel splice variants of adhesion G protein-coupled receptors. Gene 387:38–48. 10.1016/j.gene.2006.07.039 17056209

[B8] Bolliger MF, Martinelli DC, Südhof TC (2011) The cell-adhesion G protein-coupled receptor BAI3 is a high-affinity receptor for C1q-like proteins. Proc Natl Acad Sci U S A 108:2534–2539. 10.1073/pnas.1019577108 21262840 PMC3038708

[B9] Boucard AA, Ko J, Südhof TC (2012) High affinity neurexin binding to cell adhesion G-protein-coupled receptor CIRL1/latrophilin-1 produces an intercellular adhesion complex. J Biol Chem 287:9399–9413. 10.1074/jbc.M111.318659 22262843 PMC3308797

[B10] Boucard AA, Maxeiner S, Südhof TC (2014) Latrophilins function as heterophilic cell-adhesion molecules by binding to teneurins: regulation by alternative splicing. J Biol Chem 289:387–402. 10.1074/jbc.M113.504779 24273166 PMC3879561

[B11] Boxer EE, Aoto J (2022) Neurexins and their ligands at inhibitory synapses. Front Synaptic Neurosci 14:1087238. 10.3389/fnsyn.2022.1087238 36618530 PMC9812575

[B12] Cao X, Tabuchi K (2017) Functions of synapse adhesion molecules neurexin/neuroligins and neurodevelopmental disorders. Neurosci Res 116:3–9. 10.1016/j.neures.2016.09.005 27664583

[B13] Chen S, Gouaux E (2019) Structure and mechanism of AMPA receptor - auxiliary protein complexes. Curr Opin Struct Biol 54:104–111. 10.1016/j.sbi.2019.01.011 30825796 PMC6592759

[B14] Chen LY, Jiang M, Zhang B, Gokce O, Südhof TC (2017) Conditional deletion of all neurexins defines diversity of essential synaptic organizer functions for neurexins. Neuron 94:611–625.e4. 10.1016/j.neuron.2017.04.011 28472659 PMC5501922

[B15] Cortés E, Pak JS, Özkan E (2023) Structure and evolution of neuronal wiring receptors and ligands. Dev Dyn 252:27–60. 10.1002/dvdy.512 35727136 PMC10084454

[B16] Del Toro D, et al. (2020) Structural basis of teneurin-latrophilin interaction in repulsive guidance of migrating neurons. Cell 180:323–339.e19. 10.1016/j.cell.2019.12.014 31928845 PMC6978801

[B17] Einspahr JM, Tilley DG (2022) Pathophysiological impact of the adhesion G protein-coupled receptor family. Am J Physiol Cell Physiol 323:C640–C647. 10.1152/ajpcell.00445.2021 35848619 PMC9359651

[B18] Embree CM, Abu-Alhasan R, Singh G (2022) Features and factors that dictate if terminating ribosomes cause or counteract nonsense-mediated mRNA decay. J Biol Chem 298:102592. 10.1016/j.jbc.2022.102592 36244451 PMC9661723

[B19] Freitas AE, Gorodetski L, Lim WL, Zou Y (2023) Emerging roles of planar cell polarity proteins in glutamatergic synapse formation, maintenance and function in health and disease. Dev Dyn 252:1068–1076. 10.1002/dvdy.574 36780134

[B20] Fuccillo MV, Pak C (2021) Copy number variants in neurexin genes: phenotypes and mechanisms. Curr Opin Genet Dev 68:64–70. 10.1016/j.gde.2021.02.010 33756113 PMC8491281

[B21] Gomez AM, Traunmüller L, Scheiffele P (2021) Neurexins: molecular codes for shaping neuronal synapses. Nat Rev Neurosci 22:137–151. 10.1038/s41583-020-00415-7 33420412 PMC7612283

[B22] Haas KT, Compans B, Letellier M, Bartol TM, Grillo-Bosch D, Sejnowski TJ, Sainlos M, Choquet D, Thoumine O, Hosy E (2018) Pre-post synaptic alignment through neuroligin-1 tunes synaptic transmission efficiency. Elife 7:e31755. 10.7554/eLife.31755 30044218 PMC6070337

[B23] Hamann J, et al. (2015) International union of basic and clinical pharmacology. XCIV. adhesion G protein-coupled receptors. Pharmacol Rev 67:338–367. 10.1124/pr.114.009647 25713288 PMC4394687

[B24] Han Y, Cao R, Qin L, Chen LY, Tang A-H, Südhof TC, Zhang B (2022) Neuroligin-3 confines AMPA receptors into nanoclusters, thereby controlling synaptic strength at the calyx of held synapses. Sci Adv 8:eabo4173. 10.1126/sciadv.abo4173 35704570 PMC9200272

[B25] Harris KM, Weinberg RJ (2012) Ultrastructure of synapses in the mammalian brain. Cold Spring Harb Perspect Biol 4:a005587. 10.1101/cshperspect.a005587 22357909 PMC3331701

[B26] Ichtchenko K, Bittner MA, Krasnoperov V, Little AR, Chepurny O, Holz RW, Petrenko AG (1999) A novel ubiquitously expressed alpha-latrotoxin receptor is a member of the CIRL family of G-protein-coupled receptors. J Biol Chem 274:5491–5498. 10.1074/jbc.274.9.5491 10026162

[B27] Kaeser PS, Deng L, Wang Y, Dulubova I, Liu X, Rizo J, Südhof TC (2011) RIM proteins tether Ca2 + channels to presynaptic active zones via a direct PDZ-domain interaction. Cell 144:282–295. 10.1016/j.cell.2010.12.029 21241895 PMC3063406

[B28] Kakegawa W, et al. (2015) Anterograde C1ql1 signaling is required in order to determine and maintain a single-winner climbing fiber in the mouse cerebellum. Neuron 85:316–329. 10.1016/j.neuron.2014.12.020 25611509

[B29] Kandel ER (2001) The molecular biology of memory storage: a dialogue between genes and synapses. Science 294:1030–1038. 10.1126/science.1067020 11691980

[B30] Knierim AB, Röthe J, Çakir MV, Lede V, Wilde C, Liebscher I, Thor D, Schöneberg T (2019) Genetic basis of functional variability in adhesion G protein-coupled receptors. Sci Rep 9:11036. 10.1038/s41598-019-46265-x 31363148 PMC6667449

[B31] Kreienkamp HJ, Zitzer H, Gundelfinger ED, Richter D, Bockers TM (2000) The calcium-independent receptor for alpha-latrotoxin from human and rodent brains interacts with members of the ProSAP/SSTRIP/shank family of multidomain proteins. J Biol Chem 275:32387–32390. 10.1074/jbc.C000490200 10964907

[B32] Langosch D, Hoch W, Betz H (1992) The 93 kDa protein gephyrin and tubulin associated with the inhibitory glycine receptor are phosphorylated by an endogenous protein kinase. FEBS Lett 298:113–117. 10.1016/0014-5793(92)80034-E 1312018

[B33] Li C, et al. (2022) Planar cell polarity protein Celsr2 maintains structural and functional integrity of adult cortical synapses. Prog Neurobiol 219:102352. 10.1016/j.pneurobio.2022.102352 36089108

[B34] Liebscher I, et al. (2022) A guide to adhesion GPCR research. FEBS J 289:7610–7630. 10.1111/febs.16258 34729908

[B35] Lloyd BA, Han Y, Roth R, Zhang B, Aoto J (2023) Neurexin-3 subsynaptic densities are spatially distinct from Neurexin-1 and essential for excitatory synapse nanoscale organization in the hippocampus. Nat Commun 14:4706. 10.1038/s41467-023-40419-2 37543682 PMC10404257

[B36] Martinelli DC, Chew KS, Rohlmann A, Lum MY, Ressl S, Hattar S, Brunger AT, Missler M, Südhof TC (2016) Expression of C1ql3 in discrete neuronal populations controls efferent synapse numbers and diverse behaviors. Neuron 91:1034–1051. 10.1016/j.neuron.2016.07.002 27478018 PMC5017910

[B37] Matúš D, Post WB, Horn S, Schöneberg T, Prömel S (2021) Latrophilin-1 drives neuron morphogenesis and shapes chemo- and mechanosensation-dependent behavior in *C. elegans* via a trans function. Biochem Biophys Res Commun 589:152–158. 10.1016/j.bbrc.2021.12.006 34922196

[B38] Maximov A, Pang ZP, Tervo DGR, Südhof TC (2007) Monitoring synaptic transmission in primary neuronal cultures using local extracellular stimulation. J Neurosci Methods 161:75–87. 10.1016/j.jneumeth.2006.10.009 17118459

[B39] McIntire SL, Reimer RJ, Schuske K, Edwards RH, Jorgensen EM (1997) Identification and characterization of the vesicular GABA transporter. Nature 389:870–876. 10.1038/39908 9349821

[B40] Naisbitt S, Kim E, Tu JC, Xiao B, Sala C, Valtschanoff J, Weinberg RJ, Worley PF, Sheng M (1999) Shank, a novel family of postsynaptic density proteins that binds to the NMDA receptor/PSD-95/GKAP complex and cortactin. Neuron 23:569–582. 10.1016/S0896-6273(00)80809-0 10433268

[B41] Najarro EH, Wong L, Zhen M, Carpio EP, Goncharov A, Garriga G, Lundquist EA, Jin Y, Ackley BD (2012) Caenorhabditis elegans flamingo cadherin fmi-1 regulates GABAergic neuronal development. J Neurosci 32:4196–4211. 10.1523/JNEUROSCI.3094-11.2012 22442082 PMC3325105

[B42] Nozawa K, Sogabe T, Hayashi A, Motohashi J, Miura E, Arai I, Yuzaki M (2022) In vivo nanoscopic landscape of neurexin ligands underlying anterograde synapse specification. Neuron 110:3168–3185. e8. 10.1016/j.neuron.2022.07.027 36007521

[B43] O'Sullivan ML, de Wit J, Savas JN, Comoletti D, Otto-Hitt S, Yates JR, Ghosh A (2012) FLRT proteins are endogenous latrophilin ligands and regulate excitatory synapse development. Neuron 73:903–910. 10.1016/j.neuron.2012.01.018 22405201 PMC3326387

[B44] Ovando-Zambrano J-C, Arias-Montaño J-A, Boucard AA (2019) Alternative splicing event modifying ADGRL1/latrophilin-1 cytoplasmic tail promotes both opposing and dual cAMP signaling pathways. Ann N Y Acad Sci 1456:168–185. 10.1111/nyas.14198 31339586

[B45] Pederick DT, Lui JH, Gingrich EC, Xu C, Wagner MJ, Liu Y, He Z, Quake SR, Luo L (2021) Reciprocal repulsions instruct the precise assembly of parallel hippocampal networks. Science 372:1068–1073. 10.1126/science.abg1774 34083484 PMC8830376

[B46] Rosa M, Noel T, Harris M, Ladds G (2021) Emerging roles of adhesion G protein-coupled receptors. Biochem Soc Trans 49:1695–1709. 10.1042/BST20201144 34282836 PMC8421042

[B47] Sando R, Ho ML, Liu X, Südhof TC (2022) Engineered synaptic tools reveal localized cAMP signaling in synapse assembly. J Cell Biol 221:e202109111. 10.1083/jcb.202109111 34913963 PMC8685283

[B48] Sando R, Jiang X, Südhof TC (2019) Latrophilin GPCRs direct synapse specificity by coincident binding of FLRTs and teneurins. Science 363:eaav7969. 10.1126/science.aav7969 30792275 PMC6636343

[B49] Sando R, Südhof TC (2021) Latrophilin GPCR signaling mediates synapse formation. Elife 10:e65717. 10.7554/eLife.65717 33646123 PMC7954527

[B50] Sanes JR, Zipursky SL (2020) Synaptic specificity, recognition molecules, and assembly of neural circuits. Cell 181:536–556. 10.1016/j.cell.2020.04.008 32359437

[B51] Scott S, Aricescu AR (2019) A structural perspective on GABAA receptor pharmacology. Curr Opin Struct Biol 54:189–197. 10.1016/j.sbi.2019.03.023 31129381

[B52] Seufert F, Chung YK, Hildebrand PW, Langenhan T (2023) 7TM domain structures of adhesion GPCRs: what's new and what's missing? Trends Biochem Sci 48:726–739. 10.1016/j.tibs.2023.05.007 37349240

[B53] Sheng M, Kim E (2011) The postsynaptic organization of synapses. Cold Spring Harb Perspect Biol 3:a005678. 10.1101/cshperspect.a005678 22046028 PMC3225953

[B54] Shiu FH, Wong JC, Yamamoto T, Lala T, Purcell RH, Owino S, Zhu D, van Meir EG, Hall RA, Escayg A (2022) Mice lacking full length Adgrb1 (Bai1) exhibit social deficits, increased seizure susceptibility, and altered brain development. Exp Neurol 351:113994. 10.1016/j.expneurol.2022.113994 35114205 PMC9817291

[B55] Sigoillot SM, Iyer K, Binda F, González-Calvo I, Talleur M, Vodjdani G, Isope P, Selimi F (2015) The secreted protein C1QL1 and its receptor BAI3 control the synaptic connectivity of excitatory inputs converging on cerebellar purkinje cells. Cell Rep 10:820–832. 10.1016/j.celrep.2015.01.034 25660030

[B56] Silva J-P, et al. (2011) Latrophilin 1 and its endogenous ligand Lasso/teneurin-2 form a high-affinity transsynaptic receptor pair with signaling capabilities. Proc Natl Acad Sci U S A 108:12113–12118. 10.1073/pnas.1019434108 21724987 PMC3141932

[B57] Stephenson JR, Paavola KJ, Schaefer SA, Kaur B, van Meir EG, Hall RA (2013) Brain-specific angiogenesis inhibitor-1 signaling, regulation, and enrichment in the postsynaptic density. J Biol Chem 288:22248–22256. 10.1074/jbc.M113.489757 23782696 PMC3829316

[B58] Südhof TC (2013) Neurotransmitter release: the last millisecond in the life of a synaptic vesicle. Neuron 80:675–690. 10.1016/j.neuron.2013.10.022 24183019 PMC3866025

[B59] Südhof TC (2017) Synaptic neurexin complexes: a molecular code for the logic of neural circuits. Cell 171:745–769. 10.1016/j.cell.2017.10.024 29100073 PMC5694349

[B60] Südhof TC (2018) Towards an understanding of synapse formation. Neuron 100:276–293. 10.1016/j.neuron.2018.09.040 30359597 PMC6226307

[B61] Südhof TC (2021) The cell biology of synapse formation. J Cell Biol 220:e202103052. 10.1083/jcb.202103052 34086051 PMC8186004

[B62] Sugita S, Ichtchenko K, Khvotchev M, Südhof TC (1998) alpha-Latrotoxin receptor CIRL/latrophilin 1 (CL1) defines an unusual family of ubiquitous G-protein-linked receptors. G-protein coupling not required for triggering exocytosis. J Biol Chem 273:32715–32724. 10.1074/jbc.273.49.32715 9830014

[B63] Thakar S, et al. (2017) Evidence for opposing roles of Celsr3 and Vangl2 in glutamatergic synapse formation. Proc Natl Acad Sci U S A 114:E610–E618. 10.1073/pnas.1612062114 28057866 PMC5278468

[B64] Tobaben S, Südhof TC, Stahl B (2000) The G protein-coupled receptor CL1 interacts directly with proteins of the Shank family. J Biol Chem 275:36204–36210. 10.1074/jbc.M006448200 10958799

[B65] Tobaben S, Südhof TC, Stahl B (2002) Genetic analysis of alpha-latrotoxin receptors reveals functional interdependence of CIRL/latrophilin 1 and neurexin 1 alpha. J Biol Chem 277:6359–6365. 10.1074/jbc.M111231200 11741895

[B66] Trotter JH, Hao J, Maxeiner S, Tsetsenis T, Liu Z, Zhuang X, Südhof TC (2019) Synaptic neurexin-1 assembles into dynamically regulated active zone nanoclusters. J Cell Biol 218:2677–2698. 10.1083/jcb.201812076 31262725 PMC6683742

[B67] Tu Y-K, Duman JG, Tolias KF (2018) The adhesion-GPCR BAI1 promotes excitatory synaptogenesis by coordinating bidirectional trans-synaptic signaling. J Neurosci 38:8388–8406. 10.1523/JNEUROSCI.3461-17.2018 30120207 PMC6158688

[B68] Vitobello A, et al. (2022) ADGRL1 haploinsufficiency causes a variable spectrum of neurodevelopmental disorders in humans and alters synaptic activity and behavior in a mouse model. Am J Hum Genet 109:1436–1457. 10.1016/j.ajhg.2022.06.011 35907405 PMC9388395

[B69] Vizurraga A, Adhikari R, Yeung J, Yu M, Tall GG (2020) Mechanisms of adhesion G protein-coupled receptor activation. J Biol Chem 295:14065–14083. 10.1074/jbc.REV120.007423 32763969 PMC7549034

[B70] Vysokov NV, et al. (2018) Proteolytically released lasso/teneurin-2 induces axonal attraction by interacting with latrophilin-1 on axonal growth cones. Elife 7:e37935. 10.7554/eLife.37935 30457553 PMC6245728

[B71] Wang J, et al. (2021) RTN4/NoGo-receptor binding to BAI adhesion-GPCRs regulates neuronal development. Cell 184:5869–5885.e25. 10.1016/j.cell.2021.10.016 34758294 PMC8620742

[B72] Wang S, DeLeon C, Sun W, Quake SR, Roth BL, Südhof TC (2024) Alternative splicing of latrophilin-3 controls synapse formation. Nature 626:128–135. 10.1038/s41586-023-06913-9 38233523 PMC10830413

[B73] Wang CY, Liu Z, Ng YH, Südhof TC (2020) A synaptic circuit required for acquisition but not recall of social transmission of food preference. Neuron 107:144–157.e4. 10.1016/j.neuron.2020.04.004 32369733 PMC7351611

[B74] Wierenga CJ, Wadman WJ (1999) Miniature inhibitory postsynaptic currents in CA1 pyramidal neurons after kindling epileptogenesis. J Neurophysiol 82:1352–1362. 10.1152/jn.1999.82.3.1352 10482754

[B75] Zhang RS, Liakath-Ali K, Südhof TC (2020) Latrophilin-2 and latrophilin-3 are redundantly essential for parallel-fiber synapse function in cerebellum. Elife 9:e54443. 10.7554/eLife.54443 32202499 PMC7089768

[B76] Zhang X, Lin P-Y, Liakath-Ali K, Südhof TC (2022) Teneurins assemble into presynaptic nanoclusters that promote synapse formation via postsynaptic non-teneurin ligands. Nat Commun 13:2297. 10.1038/s41467-022-29751-1 35484136 PMC9050732

[B77] Zhou Q, Qin J, Liang Y, Zhang W, He S, Tissir F, Qu Y, Zhou L (2021) Celsr3 is required for Purkinje cell maturation and regulates cerebellar postsynaptic plasticity. iScience 24:102812. 10.1016/j.isci.2021.102812 34308297 PMC8283331

[B78] Zhu D, et al. (2015) BAI1 regulates spatial learning and synaptic plasticity in the hippocampus. J Clin Invest 125:1497–1508. 10.1172/JCI74603 25751059 PMC4396478

