# Supplementary figures and images for: Essential Role of Latrophilin-1 Adhesion GPCR Nanoclusters in Inhibitory Synapses
Source: J Neurosci. 2024 Apr 29;44(23):e1978232024. doi: 10.1523/JNEUROSCI.1978-23.2024 (PMC11154861; doi:10.1523/JNEUROSCI.1978-23.2024)

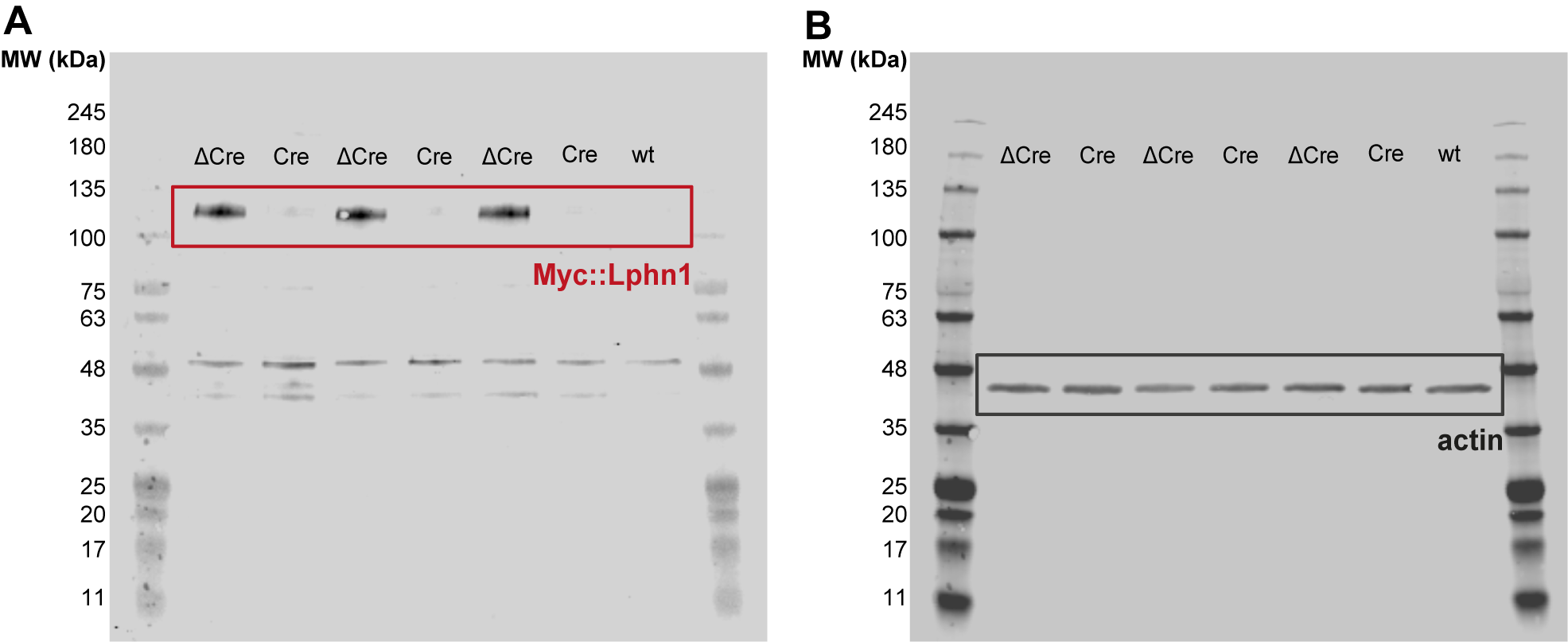

Supplement: Figure 1-1 — Full immunoblot images for the Lphn1 myc-tag epitope knockin/conditional knockout validation. (A, B) Full images of immunoblots shown in Fig. 1D. myc-tag (A) and actin staining (B) were done with distinct secondary antibodies on the same membrane. Staining for the myc tag shows a specific band at ∼115 kDa in the ΔCre condition but not in the Cre condition and the wild-type (wt) control, corresponding to the cleaved Lphn1 N terminus. Staining for actin (∼40 kDa) shows equal amount of protein has been loaded in all conditions. Unspecific bands are observed with the myc-tag immunoblotting that are not changed by the expression of Cre recombinase and are also present in wild-type controls. Download Figure 1-1, TIF file. [file jneuro-44-e1978232024-s001.tif]

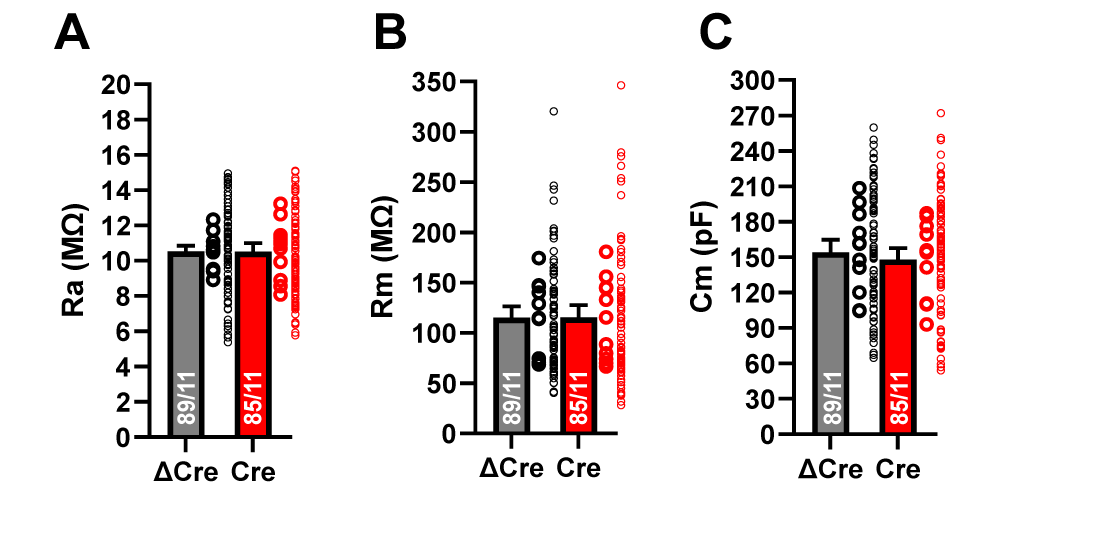

Supplement: Figure 5-1 — Intrinsic membrane properties monitored during electrophysiological recordings are unchanged by the Lphn1 deletion. Summary graphs show the mean ± SEM access resistance (A), membrane input resistance (B) and membrane capacitance (C). Numbers in bars show the number of neurons/cultures analyzed. Large and small circles next to bars indicate the mean values of each culture and the individual cell values, respectively. Statistics were done using a Mann-Whitney test on individual neuron recordings, but no significant differences have been found. Download Figure 5-1, TIF file. [file jneuro-44-e1978232024-s002.tif]

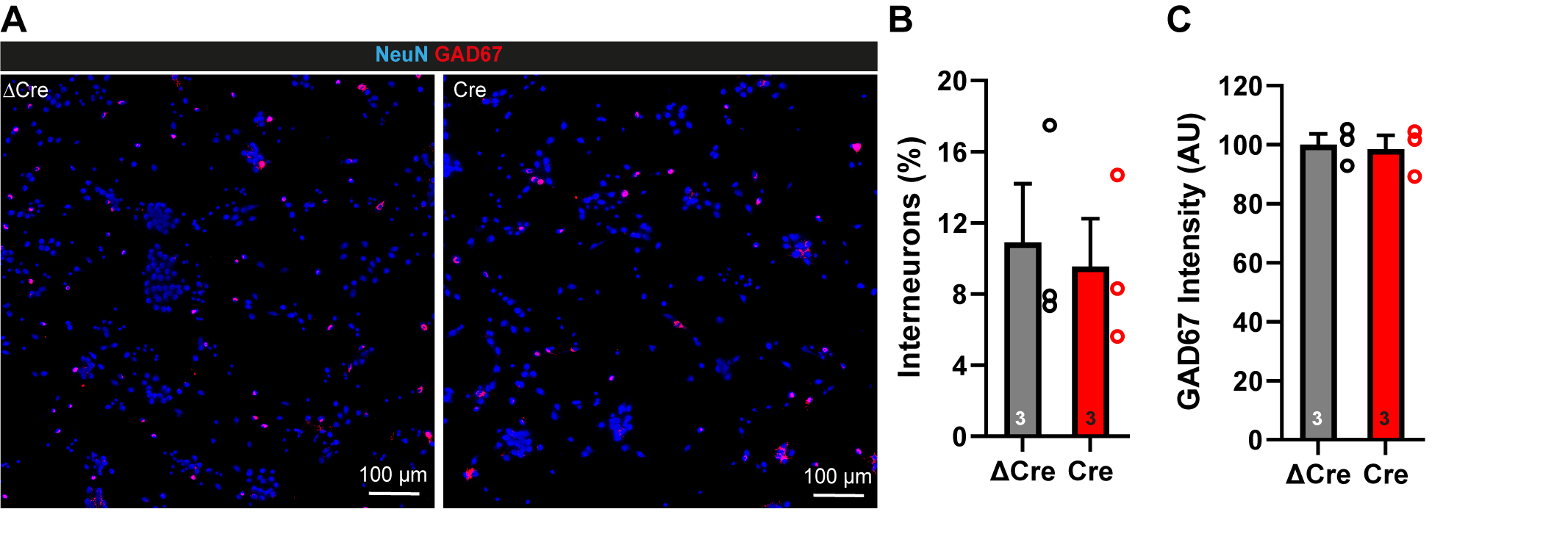

Supplement: Figure 7-1 — Inhibitory interneuron density is unchanged by the Lphn1 deletion. (A) Representative images of hippocampal neurons cultured from Lphn1 cKO mice. Neurons were infected with lentiviruses expressing active Cre recombinase or inactive mutant ΔCre and immunostained for GAD67 as a marker of inhibitory interneurons and for NeuN as a marker of all neurons. The imaging threshold was set to exclude synaptic GAD67 fluorescence, leaving only somatic fluorescence of interneurons to be analyzed. (B) Quantification of GAD67 + cells (interneurons) as a percentage of NeuN + cells reveals similar interneuron densities in Lphn1-deficient cultures compared to the control condition. (C) GAD67 staining intensity is unchanged between Cre and ΔCre expressing neurons. Multiple coverslips were scanned and pooled for each condition in each culture (n = 3). Summary graphs show means ± SEMs; circles indicate the mean values obtained in a given independent culture. Statistics were done using paired t-tests, but no significant difference has been found. Download Figure 7-1, TIF file. [file jneuro-44-e1978232024-s003.tif]
